# Monitoring Pre- and Post-Operative Immune Alterations in Patients With Locoregional Colorectal Cancer Who Underwent Laparoscopy by Single-Cell Mass Cytometry

**DOI:** 10.3389/fimmu.2022.807539

**Published:** 2022-02-03

**Authors:** Chuanyong Zhou, Zaozao Wang, Beihai Jiang, Jiabo Di, Xiangqian Su

**Affiliations:** Key Laboratory of Carcinogenesis and Translational Research (Ministry of Education), Department of Gastrointestinal Surgery IV, Peking University Cancer Hospital & Institute, Beijing, China

**Keywords:** locoregional colorectal cancer, laparoscopy, perioperative immune alterations, immunosuppression, single-cell mass cytometry

## Abstract

Surgical excision is currently the principal therapy for locoregional colorectal cancer (CRC). However, surgical trauma leads to controlled tissue damage, causing profound alterations in host immunity and, in turn, affecting post-operative outcomes. Surgery-induced immune alterations in CRC remain poorly defined. Here, single-cell mass cytometry was applied to serial blood samples collected pre-operatively, and on days 1, 3, and 7 post-operatively from 24 patients who underwent laparoscopic surgical resection of CRC to comprehensively monitor the perioperative phenotypic alterations in immune cells and dynamics of immune response. Characterization of immune cell subsets revealed that the post-operative immune response is broad but predominantly suppressive, supported by the decreases in total frequencies of circulating T cells and natural killer (NK) cells, as well as decreased HLA-DR expression on circulating monocytes. The proportion of T cells significantly decreased on day 1 and recovered to the pre-surgical level on day 3 after surgery. The frequency of monocytes was significantly elevated on day 1 after surgery and declined to baseline level on day 3. NK cells temporarily contracted on post-operative day 3. T cells, monocytes, DCs, NK cells, and B cells were partitioned into phenotypically different single-cell clusters. The dynamics of single-cell clusters were different from those of the bulk lineages. T cell clusters in the same response phase fluctuate inconsistently during the perioperative period. Comparing to the baseline levels, the frequencies of CD11b(+)CD33(+)CD14(+)CD16(−) classical monocytes expanded followed by contraction, whereas CD11b(+)CD33(+)CD14(high)CD16(low) intermediate monocytes remained unchanged; HLA-DR expression in monocytes were significantly reduced; the frequencies of intermediate CD56(bright)CD16(+) NK cell subsets increased; and the percentage of memory B lymphocytes were elevated after surgery. Post-operative pro- and anti-inflammatory cytokines were both altered. Furthermore, perioperative immune perturbations in some of the cell subsets were unrecovered within seven days after surgery. Chronological monitoring major immune lineages provided an overview of surgery-caused alterations, including cell augments and contractions and precisely timed changes in immune cell distribution in both innate and adaptive compartments, providing evidence for the interaction between tumor resection and immune modulation.

## Introduction

Colorectal cancer (CRC) is one of the leading causes of cancer-related deaths worldwide, with a 5-year survival rate of < 65% (1). After primary treatment, tumor-free patients may subsequently develop locoregional recurrence (18%), distant metastasis (78%), or both (4%) (2). Surgical excision is currently the principal therapy for locoregional CRC. Although surgical resection of solid tumors provides a significant opportunity for recovery in many patients, surgical trauma leads to controlled tissue damage, which may cause profound alterations in host immunity.

The complex immune response to surgical stress has prompted a long-standing interest in elucidating the interaction between tumor resection, immune modulation, and clinical outcomes. Both innate and adaptive immunity are disrupted by surgical stress. Within hours after surgery, the level of proinflammatory cytokines increases, the activity of natural killer (NK) cells, total lymphocytes, and dendritic cells (DCs) is impaired for one week, and cellular immunity is restored within two weeks after surgery (3). Following surgical trauma, temporary inflammation occurs, followed by a longer immunosuppression duration (4). The role of immunosuppression is to suppress the proinflammatory response and reduce the risk of autoimmune disease and tissue necrosis (5). Post-operative immunosuppression has been reported in surgeries of multiple cancer types, which may awake dormant cancer cells leading to rapid local and distant tumor recurrences or metastases, including CRC (3, 6). The efficacy of cellular immunity during surgery is related to disease-free survival (7). The improved preservation of cellular immunity has been related to a lower incidence of local recurrence and distant tumor metastasis (8).

Manipulation of perioperative immune responses has the potential to improve patient outcomes (9–11). As the suppression and activation of immune cell clusters are of therapeutic significance, a deep characterization of the perioperative immune response is of great importance. Therefore, a complete understanding of the altered immune function in this setting is imperative. However, limited information is available regarding the perioperative immunological effects of laparoscopic surgery after CRC excision, and investigations mainly focus on perioperative cytokine production and changes in immune cell count after surgery (12–14). As none of these studies have measured immune functional responses at the single-cell level, specific perioperative immune signatures have been functionally and phenotypically overlooked.

In the current study, we monitored the peripheral immune perturbations during the pre-operative and early post-operative periods of 24 CRC patients who underwent radical laparoscopic surgeries by single-cell mass cytometry-based precise phenotyping of immune cell subsets. Using high-sensitivity ProcartaPlex cytokine analysis, we assessed the *in vivo* perioperative cytokine production. This study, therefore, allows the extraction of surgery-specific single-cell immune alterations of patients with CRC and to systemically elucidate perioperative immune cell diversity and dynamics.

## Materials and Methods

### Human Specimens

Peri-operative blood samples were obtained from patients with right-sided, left-sided colon or rectal cancer who underwent resection surgery at the Department of Gastrointestinal Surgery IV, Peking University Cancer Hospital & Institute, after obtaining informed consent. Left-sided colon cancer consists of cancers of the descending and sigmoid colon, right-sided colon cancer consists of cancers of the caecum and the transverse colon (15). The protocols were reviewed and approved by the Research Ethics Committee of Peking University Cancer Hospital & Institute, Beijing, China (No. 2019KT33). We collected one pre-operative (day 0) and three post-operative time points (days 1, 3, and 7) from each patient during the hospitalization period of 24 patients. Inclusion criteria were: 1) being scheduled for laparoscopic CRC resection, 2) an age 18–80 years, 3) willingness to sign informed consent, and 4) having not received neoadjuvant chemo-, radio-, or targeted- therapy. The exclusion criteria were: 1) having developed post-operative complications or infections and 2) any disease or medication that may affect the immune system. Clinicopathological characteristics of patients (**Supplementary Table 1**) were determined based on the National Comprehensive Cancer Network (NCCN) guidelines for colon cancer (Version 4.2020) and rectal cancer (Version 6.2020).

### Sample Collection, Storage, and PBMC Isolation

Blood was collected into blood collection tubes (BD Vacutainer^®^, Becton, Dickinson and Company, Franklin Lakes, NJ, USA) containing ethylenediaminetetraacetic acid (EDTA). Peripheral blood mononuclear cells (PBMCs) were collected *via* density-gradient centrifugation using human PBMC isolation buffer (Solarbio^®^ Life Science & Technology, Beijing, China), and red blood cells were lysed for 5 min at 21°C using a red blood cell lysis Buffer (Solarbio^®^). For mass cytometry analysis, PBMCs were stained for 2 min in 5 μM Cell-ID™ cisplatin (Fluidigm Corporation, South San Francisco, CA, USA), fixed for 10 min with Ca2^+^- and Mg^2+^-free phosphate-buffered saline (PBS; Hyclone, South Logan, UT, USA) containing 1.6% paraformaldehyde (PFA, Sigma-Aldrich, St. Louis, MO, USA), and then stored at −80°C. For immunofluorescence flow cytometry, isolated PBMCs were stored in liquid nitrogen without fixation.

Blood was also collected into BD Vacutainer^®^ SST™ tubes, and serum was purified *via* centrifugation at 2500 rpm for 10 min at 4°C. Purified serum was stored at −80°C.

### Antibodies and Antibody Labeling for Mass Cytometry

A total of 40 antibodies were designed to facilitate the characterization of immune cell phenotypes in PBMCs. Purified primary antibodies were purchased from BioLegend (**Supplementary Table 2**). Antibody labeling with the indicated metal isotopes was performed using the Maxpar^®^ X8 Multimetal Labeling Kit (Fluidigm) according to the manufacturer’s instructions. Conjugated antibodies were titrated to determine the optimal concentration before the experiments.

### Cell Staining and Mass Cytometry

Samples were thawed in cell staining buffer (CSB, Fluidigm) on the labeling day. PBMCs from the same patient at different time points (baseline and post-operative days 1, 3, and 7) were barcoded with CD45-163Dy, CD45-106Cd, CD45-112Cd, and CD45-114Cd, respectively, for 30 min at 21°C. After washing with CSB, PBMCs from the same patient were washed stringently, pooled, and labeled with an antibody cocktail containing 36 cell surface markers for 30 min. Cells were then washed with CSB and resuspended in Cell-ID™ Intercalator-Ir (Fluidigm) 1:1000 diluted in Fix and Perm Buffer (Fluidigm) and incubated at 4°C overnight. The cells were then washed four times with CSB and then twice in deionized water. Immediately before data acquisition, the cells were resuspended at 1 × 10^6^ cells/mL in deionized water containing 10% EQ Four Element Calibration Beads (Fluidigm). The cells were also filtered through a 35-μm membrane filter (BD Biosciences, San Jose, CA, USA) before testing.

Data acquisition was performed with Helios™ (Fluidigm) at a rate of 300–400 events per second. A total of 3~5 × 10^5^ events were collected from each patient. Data were normalized using the normalization passport EQ-P13H2302_ver2 for each experiment.

### Mass Cytometry Data Analysis

For data pre-processing, CD45-barcoded files (.fcs) were first uploaded on Cytobank (Cytobank Inc., Santa Clara, CA, USA) for manual Boolean gating. The normalization beads, dead cell debris, and cell clusters were excluded, and debarcoded DNA+ cisplatin(low) CD45+ events were exported for subsequent high-dimensional analyses.

Then, Fcs. files were imported into R (version 4.0.3) for downstream analysis using the flowCore package (version 2.0.1). Marker intensities were arcsinh transformed with a cofactor of 5 [x_transf = asinh(x/5)] to make the signal distribution more symmetric and the range of expression more comparable.

The Fast Fourier Transform (FFT)-accelerated Interpolation-based t-Stochastic Neighborhood Embedding (FIt-SNE, version 1.2.1) algorithm was implemented to reduce and visualize the high-dimensional marker expression data in two dimensions (16). For each sample, a total of 10^4^ cells were randomly selected for the FIt-SNE analysis. All cells were used if fewer than 10^4^ cells were detected in the sample. To visualize the expression of markers on FIt-SNE maps, we excluded the highest percentile, and the maximum intensity was set to the 99^th^ percentile. Data from all samples were divided by this value to obtain signal intensities in the range of 0 and 1 for each channel. The data are displayed using the ggplot2 R package (version 3.3.3).

Clustering analysis was performed using the R PhenoGraph package (version 0.99.1), and PhenoGraph was run on all samples simultaneously. To identify the major cell lineages in the datasets, PhenoGraph was run with the parameter k set to 40. To identify T cell subsets, PhenoGraph was run with the parameter k = 25. To identify monocyte and DC clusters, PhenoGraph was run with the parameter k = 20. To identify NK cell clusters, PhenoGraph was run with k set to 30. Heatmaps were displayed in R using the heatmap function in the Complex Heatmap package (version 2.4.3). For hierarchical clustering, Euclidean correlation was used to calculate the pairwise distances between samples. The dendrogram was extracted and plotted using the R ggdendro package (version 0.1.22).

### Cell Processing and Fluorescent Flow Cytometry

To determine the perioperative Treg alteration, PBMCs were thawed and washed with PBA (PBS containing 0.1% BSA and 0.05% sodium azide). Then, cells were incubated with the following antibodies at 4°C for 30 min: Anti-Human CD4-Alexa Fluor 488 (clone PRA-T4, eBioscience™, Thermo Fisher Scientific, Waltham, MA, USA), Anti-Human CD127-APC (clone eBioRDR5, eBioscience™), and Anti-Human CD25-PE (clone BC96, eBioscience™). Before flow cytometric analysis, the cells were washed and resuspended in PBA. Cells were then examined with a cytometryLEX flow cytometer (Beckman Coulter, Miami, FL, USA), and data analysis was performed with Cytobank.

### Cytokine Measurement

Perioperative cytokine secretion in serum was determined using the ProcartaPlex™ Human High-sensitivity Panel (9-plex, Thermo Fisher Scientific) according to the manufacturer’s instructions. The serum concentrations of interferon (IFN)-γ, interleukin (IL)-1β, IL-2, IL-4, IL-6, IL-10, IL-12p70, IL-17A, and tumor necrosis factor (TNF)-α were measured. A Luminex 200 system (Luminex Corporation, Austin, TX, USA) was used for cytokine detection.

### Statistics

In this study, immune cell alterations were treated as proportions. The Krustal-Wallis analysis with *post-hoc* Dunn test was used to compare differences between the mean frequencies of cell clusters at different timepoints or tumor locations. P-values were adjusted with the Benjamini-Hochberg method. The Wilcoxon test was applied to calculate differences between the means of cell clusters in patients with stage II vs stage III tumors, patients < 60 vs ≥ 60 years, and smokers vs non-smokers. Statistical significance was set at p < 0.05.

## Results

### High-Dimensional Single-Cell Profiling of Perioperative Immune Compositions in CRC

We performed a large-scale profiling of 96 prospectively collected PBMC samples from 24 patients diagnosed with CRC by single-cell mass cytometry. The baseline clinicopathological characteristics of the patients are presented in **Supplementary Table 1**.

**Figure 1:**
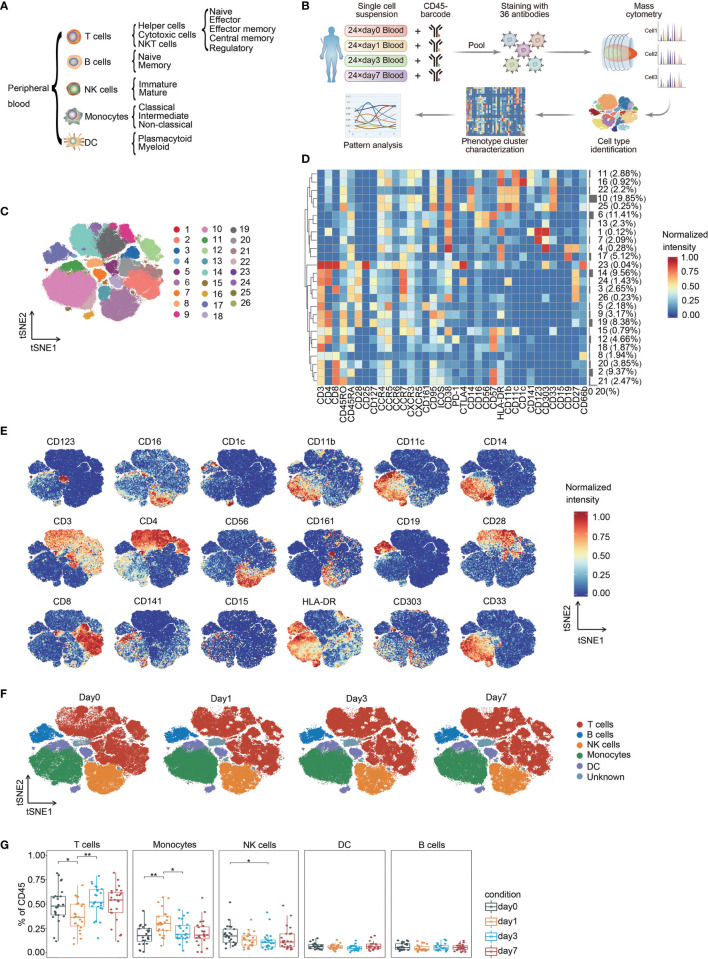
Identification of the main immune components in CRC. **(A)** Immune cell lineages and respective subpopulations of interest for comprehensive immunophenotyping. **(B)** Experimental strategy used in this study. **(C)** FIt-SNE of immune cells colored by PhenoGraph clusters. **(D)** Heatmap showing the normalized expression of the markers for PhenoGraph clusters. Clusters are grouped by surface marker expression profiles. The cluster numbers and relative frequencies are exhibited on the right. **(E)** FIt-SNE plots of normalized marker expression for 10,000 cells randomly selected from all patients. **(F)** FIt-SNE plots highlighting the distribution of major cell lineages. **(G)** Boxplots showing the frequencies of each immune cell lineage at different time point. *p < 0.05, **p < 0.01 by Krustal-Wallis analysis with *post-hoc* Dunn test. P-value adjusted with the Benjamini-Hochberg method. NK, natural killer; DC, dendritic cell.

We built a comprehensive single-pass immunophenotyping panel to identify major immune cell lineages and their subsets (**Figure 1A**). PBMCs from different perioperative timepoints were barcoded with anti-CD45 antibodies conjugated to distinct metal epitopes before the samples were pooled. Pooled samples were stained with our panel and analyzed by single-cell mass cytometry (**Figure 1B**). This approach allows for the simultaneous profiling of cells from different time points from the same patient (17), allowing the determination of surgery-related changes in immune compartments.

The complete dataset contained over 33 million single-cell profiles, and the non-beads, single, live, and debarcoded CD45(+) cell count from each sample that passed pre-processing is summarized in **Supplementary Figure 1**. Sequential manual gating was applied to confirm the major immune cell lineages and their subpopulations (**Supplementary Figure 2**). All immune cell populations of interest could be identified, and the calculated frequencies were within known ranges (18).

### Stratification of Immune Perturbations During Perioperative Period in CRC

To generate a comprehensive view of perioperative immunity, the FIt-SNE algorithm was employed to generate two-dimensional maps from high-dimensional data (16). The PhenoGraph clustering algorithm was applied to explicitly identify and partition cells into different phenotypes (19). This analysis identified 26 distinct metaclusters and enabled visualization of the major immune cell types of interest within the CD45(+) compartment (**Figures 1C–E**). We were able to assign 97.23% of the pre-gated cells to a specific immune cell lineage.

T cells and monocytes comprised the largest populations in the mononuclear fraction of peripheral blood, followed by NK cells (**Figures 1F, G**, **Supplementary Figure 3**). The proportion of T cells significantly decreased on day 1 and recovered to the pre-surgical level on day 3 after surgery. The frequency of monocytes was significantly elevated on day 1 after surgery and declined to baseline level on day 3. NK cells temporarily contracted on day 3 after surgery (**Figure 1G**). Previous studies have shown a similar trend of immune cell fluctuation around the perioperative period or trauma (13, 20–22). Moreover, we performed hierarchical clustering to explore the heterogeneity of immune cell signatures across patients during the perioperative period, ordered by compositional similarity. This approach indicates considerable inter-patient variation in the frequency of immune cells associated with surgery in patients with CRC (**Supplementary Figure 4**).

### In-Depth Characterization of T Cell Subpopulations

**Figure 2:**
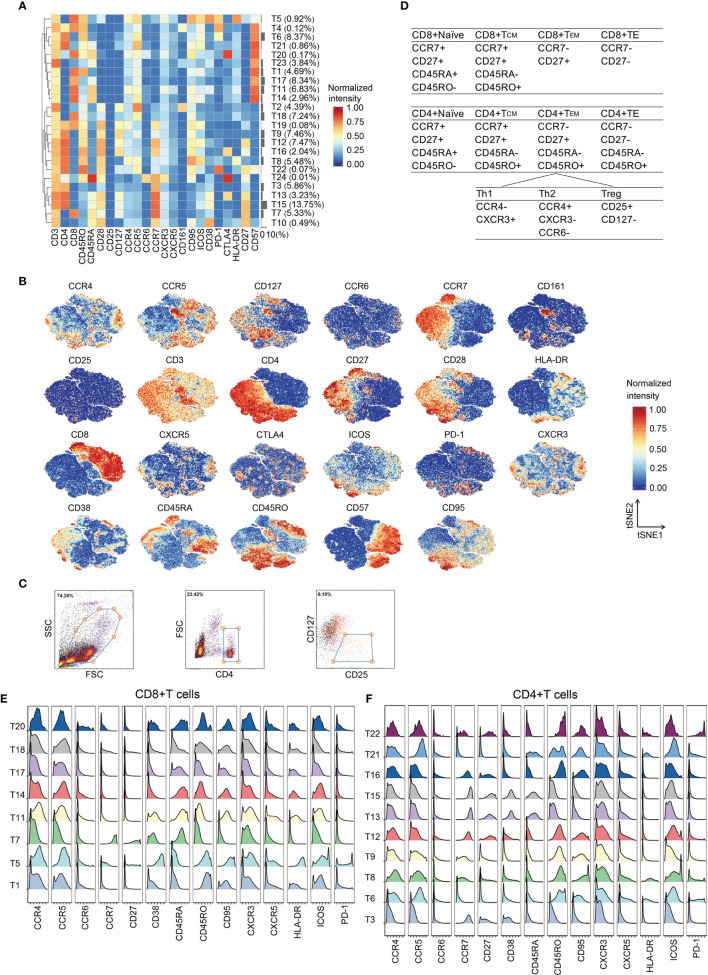
In-depth characterization of the T cell compartment. **(A)** Heatmap showing normalized expression of the markers for the 24 T cell clusters. Clusters are grouped by marker expression patterns. The cluster numbers and relative frequencies are displayed on the right. **(B)** FIt-SNE plots of normalized marker expression for 10,000 T cells randomly selected from all patients. **(C)** Exemplary identification of Treg from a representative patient. PBMCs were labeled with the indicated antibodies, examined by flow cytometry, and then analyzed by Cytobank. The Tregs were identified through the indicated strategy. **(D)** Surface marker combinations used to identify T cell subsets. **(E, F)** Histograms showing the expression of indicated functional markers on CD8(+) T cells **(E)** and CD4(+) T cells **(F)**.

To map the T cell phenotype more exhaustively, we performed additional PhenoGraph analyses (**Figure 2A**) focused on T cell metaclusters based on the initial analysis (**Figure 1D**). Heterogeneity in marker expression was assessed at the single-cell level using the FIt-SNE (**Figure 2B**). Using this approach, we identified ten CD4(+) phenotypes, eight CD8(+) phenotypes, two CD4/CD8 double-positive phenotypes, three CD4/CD8 double-negative phenotypes, and a NKT phenotype (**Figure 2A**). Regulatory T cells (Tregs) were not partitioned by PhenoGraph; thus, their proportions were determined by fluorescent flow cytometry in parallel. **Figure 2C** shows the gating strategy.

Phases of T cell responses comprise of antigen recognition, activation, clonal expansion, differentiation, and effector functions (23). Upon antigen activation and appropriate ligand co-stimulation, naïve T cells differentiate into effector T cells (24). Effector T cells proliferate and eliminate immunological threats (24). Following antigen clearance, effector cells contract into memory status (24). CD4(+) and CD8(+) T cells at different phases of adaptive immune responses were identified according to the differential expression patterns of CCR7, CD27, CD45RA, and CD45RO (25, 26) (**Figure 2D**). Most CD8(+) T cell clusters presented an effector phenotype (T1, T5, T11, T14, T17, T18, and T20), whereas only one naïve phenotype (T7) was observed (**Figures 2A, E**). T5 expressing high levels of CD38, HLA-DR, CD95, PD-1 and ICOS is a cluster of activated CD8+ T cells. CD4(+) T cell clusters exhibited naïve (T13 and T15), effector (T6, T8, T9, and T21), central memory (T12 and T16), and effector memory (T22) phenotypes (**Figures 2A, F**). T8 comprises activated CD4(+) T cells.

### T Cell Clusters in the Same Response Phase Fluctuate Inconsistently Surrounding Surgery

**Figure 3:**
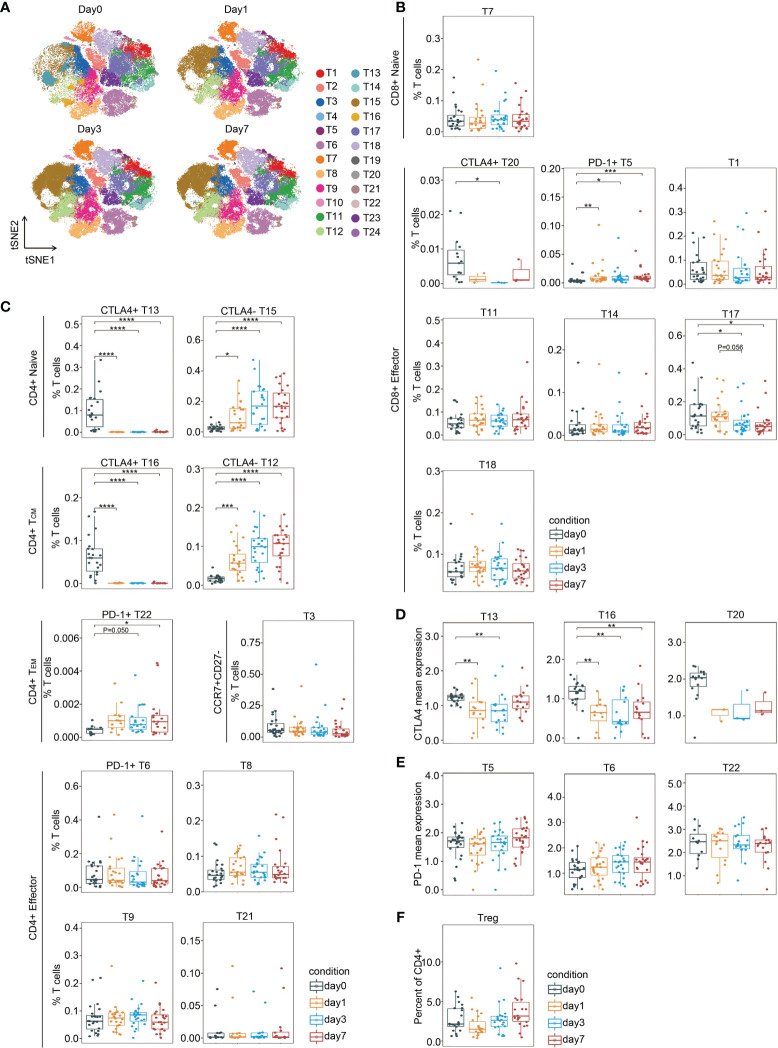
T cell fluctuation during perioperative period. **(A)** FIt-SNE plots highlighting the distribution of T cells at different time points. **(B, C)** Boxplots showing the frequencies of the CD8(+) T cell clusters **(B)** and the CD4(+) T cell clusters **(C)** during the perioperative period. **(D, E)** Boxplots showing the mean expression levels of CTLA-4 **(D)** and PD-1 **(E)** in T cells during the perioperative period. **(F)** Boxplots showing the frequencies of Treg cells during the perioperative period. *p < 0.05, **p < 0.01, ***p < 0.001, ****p < 0.0001 by Krustal-Wallis analysis with *post-hoc* Dunn test. P-value adjusted with the Benjamini-Hochberg method.

PhenoGraph clustering across all patients and across perioperative timepoints revealed that multiple T lymphoid subsets were perturbed by resection surgery for CRC (**Figure 3A**). The perioperative fluctuation trends of T cell subpopulations within the same immune response phases were differential (**Figures 3B, C**). The frequency of CD8(+)CTLA-4(+) cluster (T20) significantly decreased on day 3 after surgery comparing to the pre-surgical level and remained low on day 7, except for one outlier, whereas the frequency of CD8(+)PD-1(+) phenotype (T5), significantly increased after surgery and remained high until the last day of examination (**Figure 3B**). The frequencies of CD4(+)CTLA-4(+) clusters (T13 and T16) remarkably decreased after surgery, whereas the frequencies of CD4(+)CTLA-4(-) clusters (T12 and T15) were significantly elevated in comparison to the baseline. Interestingly, T13 and T15 were naïve cells, and T12 and T16 comprised T_CM_ cells. Of the PD-1(+) CD4(+) T cell subset, the frequency of T_EM_ cells (T22) showed a significant increase after surgery, whereas the effector subset T6 remained unchanged in comparison to the presurgical level (**Figure 3C**). Moreover, in comparison to the presurgical level, CTLA-4 expression in the CD4(+) compartments was dramatically downregulated after surgery (**Figure 3D**), while PD-1 expression levels were not affected (**Figure 3E**). Collectively, these data indicate differentially regulated T cell subpopulations upon surgical stress in patients with CRC. After gastric cancer surgery, PD-1(+)CD4(+) T cells significantly increased, reaching a maximum on day 1 and remaining elevated on day 3, whereas PD-1(+)CD8(+) T cells reached a maximum on day 7 (27). Elevated PD-1 and CTLA-4 expressing CD8(+) T cell frequency have been reported in perineural squamous cell carcinoma (28). Furthermore, the frequency of Tregs remained unchanged (**Figure 3F**).

### CD11b(+)CD33(+)CD14(+)CD16(−) Classical Monocytes Showed Expansion Followed by Contraction After Surgery

Monocytes and DCs are antigen-presenting cells. Monocytes are the first immature cell type of mononuclear phagocytes that enter the peripheral blood after leaving the marrow; once they settle in tissues, they mature and become macrophages (29). As monocytes are precursors for potent antigen-presenting DCs in peripheral organs, especially during inflammation (29), we analyzed these cells together.

**Figure 4:**
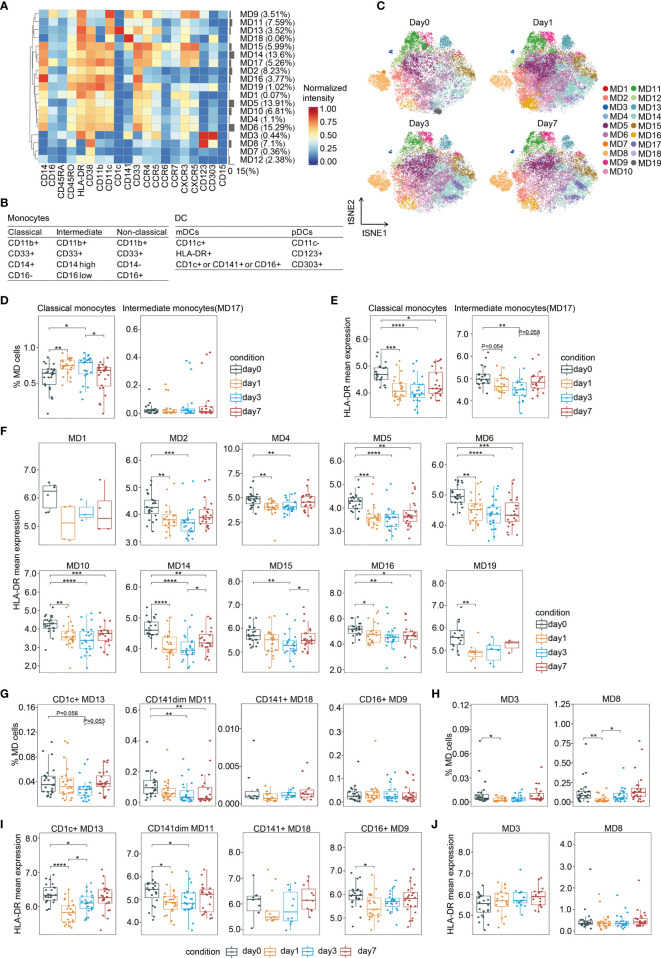
Characterization of perioperative monocytes and DCs. **(A)** Heatmap showing normalized expression of the markers for the 19 monocytes and DC clusters. Clusters are grouped by marker expression patterns. The cluster numbers and relative frequencies are displayed on the right. **(B)** Surface marker combinations used to identify monocytes and DC subsets. **(C)** FIt-SNE of monocytes and DCs colored by PhenoGraph clusters at different time points. **(D)** Boxplots showing the frequencies of the classical and intermediate monocytes during the perioperative period. **(E)** Boxplots showing the mean expression levels of HLA-DR in monocytes during the perioperative period. **(F)** Cluster-specific analysis of HLA-DR expression in monocytes shown by boxplots. **(G, H)** Boxplots showing the frequencies of the mDCs **(G)** and the pDCs **(H)** during the perioperative period. **(I, J)** Boxplots showing the cluster-specific mean expression of HLA-DR in mDCs **(I)** and in pDCs **(J)** during the perioperative period. *p < 0.05, **p < 0.01, ***p < 0.001, ****p < 0.0001 by Krustal-Wallis analysis with *post-hoc* Dunn test. P-value adjusted with the Benjamini-Hochberg method.

As shown in **Figure 4A** and **Supplementary Figure 5A**, monocytes could also be subdivided into various functional subsets by PhenoGraph and FIt-SNE based on the initial analysis (**Figure 1D**). Monocytes were characterized mainly according to their expression of CD14 and CD16 (30). All monocyte clusters expressed myeloid markers, including CD11c, CD11b, and CD33, as well as the activation markers HLA-DR and CD38 (**Figures 4A, B**). The major monocyte subset is CD11b(+)CD33(+)CD14(+)CD16(−) (MD1, MD2, MD4, MD5, MD6, MD10, MD14, MD15, MD16, and MD19), which accounts for approximately 93% of the total monocyte population, and is referred to as classical monocytes (30). The expression profiles of several surface markers differed among the 10 classical monocyte subclusters (**Figure 4A**). One intermediate monocyte group was identified (MD17), bearing surface markers CD11b(+)CD33(+)CD14(high)CD16(low) (**Figures 4A, B**). However, non-classical monocytes, which is CD11b(+)CD33(+)CD14(−)CD16(+) (31), were not observed. In fact, it has been shown that CD16(+) monocytes drive the expansion of T cells in a mixed lymphocyte reaction better than CD16(−) monocytes (32).

Surgical stress-induced monocyte fluctuations were observed during the perioperative period (**Figure 4C**). The frequency of total classical monocytes significantly increased on day 1, remained high on day 3 after surgery, and expeditiously dropped to the pre-operative baseline level on day 7. In contrast, the proportion of intermediate monocytes was not altered (**Figure 4D**). A previous study at the single-cell level showed that CD14(+) monocytes increased 2.4-fold and 1.8-fold on days 1 and 3 after surgery, respectively (33). Cluster-specific analysis of cell frequency changes revealed significant variation in one out of the 10 classical monocyte clusters during the perioperative period (**Supplementary Figure 5B**).

### Reduced HLA-DR Expression on CD11b(+)CD33(+)CD14(+)CD16(−) Monocytes After Surgery

Human leukocyte antigen class II (HLA-DR) molecules on antigen-presenting cells display cell-associated antigens for recognition by T cells. Thus, we assessed the level of its expression on circulating monocytes. As shown in **Figure 4E**, in comparison to the baseline levels, there were significant reduction in the expression of HLA-DR in classical and intermediate monocytes on day 1 and day 3, respectively. On day 7 after surgery, the down-regulation of HLA-DR expression in classical monocytes persist, while that in intermediate monocytes recovered to presurgical level. Cluster-specific analysis revealed that except for MD1, all the other monocyte clusters showed a reduction in HLA-DR during the perioperative period (**Figure 4F**). Reduced HLA-DR expression in five out of the ten clusters of classical monocytes persisted lower than pre-surgical level on day 7, whereas HLA-DR reduction in the other clusters recovered to pre-operative levels on days 3 or 7 (**Figure 4F**). Downregulated HLA-DR expression in circulating monocytes decrease antigen presentation (34). Monocytes bearing surface markers CD14(+)HLA-DR(low) have emerged as important mediators of immunoparalysis (35). The expansion of these cells after major abdominal surgery has been reported previously (20). Collectively, these data suggest that CD11b(+)CD33(+)CD14(+)CD16(−)HLA-DR(low) classical monocytes might be elevated to exert an immunosuppressive effect after surgery.

### DC Subset Balance Was Altered During Perioperative Period

DCs are professional antigen-presenting cells that initiate and direct immune responses. DCs contain two subsets of distinct origin, function, and localization, namely myeloid DCs (mDCs) and plasmacytoid DCs (pDCs). Additionally, mDCs are CD11c(+)HLA-DR(+) and can be further divided based on the expression of CD1c, CD16, and CD141 (36), whereas pDCs are characterized by CD11c(-)CD123(+)CD303(+) (37). As shown in **Figures 4A, B**, MD9 contained CD16(+) mDC, MD13 was CD1c(+) mDC, MD11 and MD18 were CD141(+) mDC, and MD3 and MD8 represented pDCs.

The frequencies of both mDC and pDC subsets were altered by surgery-induced stress. The prevalence of CD141(dim) mDC (MD11), significantly declined on day 3 after surgery, and remained lower than the pre-surgical level during the follow-up period (**Figure 4G**). pDCs are proficient in antiviral immunity by generating type I interferons (38). Our data showed that the frequencies of both pDC subpopulations rapidly decreased on day 1 after surgery and recovered to the pre-surgical level on day 3 (**Figure 4H**). Similarly, a statistically significant decline in the mean number of CD123(+) DCs has been reported in patients who underwent gastrointestinal tract cancer surgery (39). Cluster-specific analysis revealed three out of the four mDC subpopulations showed a temporarily significant reduction in HLA-DR and recovery to presurgical levels on days 3 or 7 after surgery (**Figure 4I**). In contrast, HLA-DR expression in pDCs remained unchanged (**Figure 4J**).

### The Frequency of an Intermediate CD56(bright)CD16(+) NK Cell Subset Increased After Surgery

**Figure 5:**
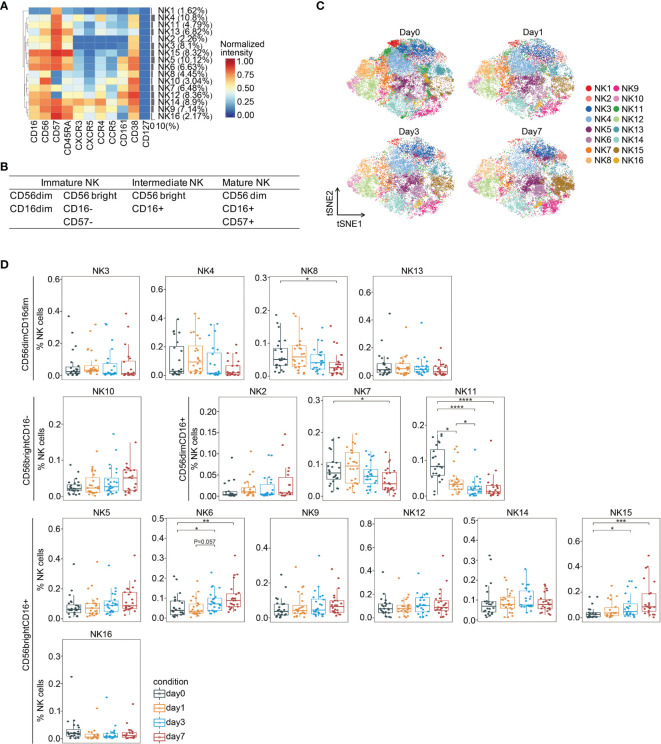
In-depth analysis of NK cell subsets during the perioperative period. **(A)** Heatmap showing normalized expression of the markers for the 16 NK cell clusters. Clusters are grouped by marker expression patterns. The cluster numbers and relative frequencies are displayed on the right. **(B)** Surface marker combinations used to identify NK cell subsets. **(C)** FIt-SNE plots highlighting the distribution of NK cells at different time points. **(D)** Boxplots showing the frequencies of NK cell clusters during the perioperative period. *p < 0.05, **p < 0.01, ***p < 0.001, ****p < 0.0001 by Krustal-Wallis analysis with *post-hoc* Dunn test. P-value adjusted with the Benjamini-Hochberg method.

In addition to measuring adaptive immune responses, we also analyzed NK cells, which are cytotoxic lymphocytes and a primary component of innate immunity. Low NK cell cytotoxicity during the perioperative period is associated with increased cancer recurrence and mortality in cancer (40, 41). There are six stages of NK cell development (42). Multiple NK cell subsets at stages 2b to 6 were identified by PhenoGraph and FIt-SNE based on the absence and presence of CD56 and CD16 (**Figures 5A, B** and **Supplemental Figure 6**). We detected immature CD56(dim)CD16(dim) (NK3, NK4, NK8, and NK13), immature CD56(bright)CD16(−) (NK10), and mature CD56(dim)CD16(+) (NK2, NK7, and NK11) NK cells (**Figure 5A**), belonging to stages 2b and 3, stage 4, and stages 5 and 6, respectively. Moreover, we also observed CD56(bright)CD16(+) cells (NK5, NK6, NK9, NK12, NK14, NK15, and NK16), which have been previously reported as a phenotypic intermediary profile between CD56(bright)CD16(−) and CD56(dim)CD16(+) NK cells (43).

We observed significant changes in the frequencies of several NK cell subsets during the perioperative period (**Figures 5C, D**). Comparing to the baseline level, the frequency of an immature cluster (NK8) was significantly reduced on day 7 after surgery. The frequencies of two mature clusters (NK7 and NK11) significantly declined after surgery. The proportion of two intermediate clusters (NK6 and NK15), both increased on day 3 and remained significantly higher than the pre-surgical level on day 7. Collectively, these data indicate that immature and mature NK cells are suppressed after surgery, whereas intermediate NK cells are activated, which has been reported playing a role in anti-tumor cytotoxicity (44). A previous study showed impaired post-operative NK cell cytotoxicity after rectal tumor surgery from days 1 to 7 (45).

### Memory B Lymphocytes Are Elevated After Surgery

We used CD19 to identify B cells and CD27 to determine their activation status (46). As shown in **Figure 1D**, both naïve [CD19(+)CD27(−)] and memory [CD19(+)CD27(+)] B cells were detected in the peripheral blood of patients with CRC after tumor resection. Although total B cell remained unchanged after surgery, we found that the frequency of memory B cells were significantly elevated on day 7 after surgery, whereas naïve B cells remained unchanged comparing to the pre-surgical level (**Supplementary Figure 7A**).

### Pro- and Anti-Inflammatory Cytokines Are Produced Following Laparoscopic Surgery in CRC

**Figure 6:**
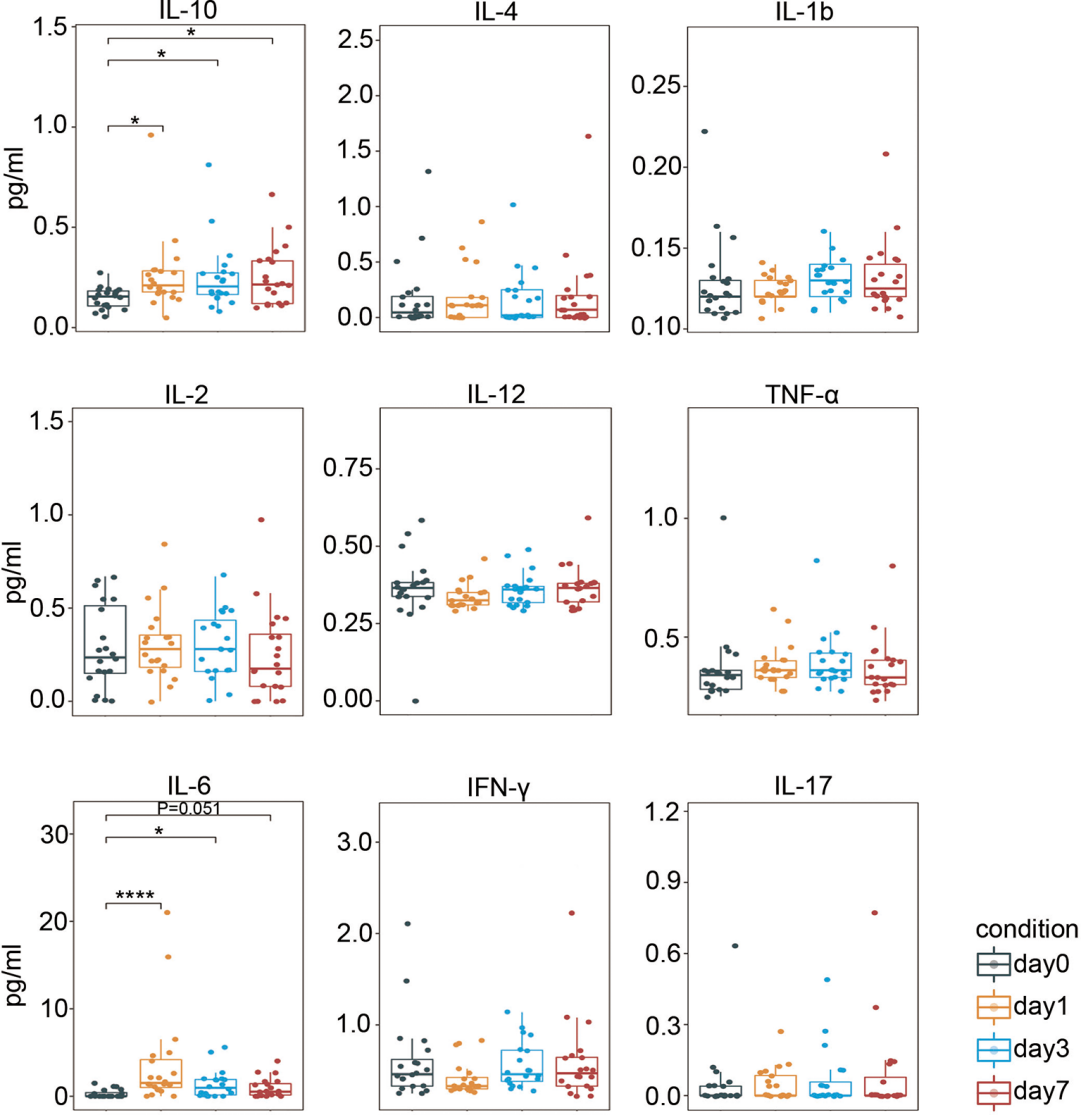
Perioperative serum cytokine levels. Boxplots showing the perioperative serum cytokine secretion measured by ProcartaPlex™. IL, interleukin; IFN-γ, interferon-γ; TNF-α, tumor necrosis factor-α. *p < 0.05, ****p < 0.0001 by Krustal-Wallis analysis with *post-hoc* Dunn test. P-value adjusted with the Benjamini-Hochberg method.

We then determined the serum cytokine levels using a highly sensitive ProcartaPlex™ cytokine panel. As shown in **Figure 6**, secretion of proinflammatory cytokine IL-6 (47) rapidly increased on day 1 after surgery, and its level remained higher than the baseline level. Meanwhile, the level of an anti-inflammatory cytokine, IL-10 (48), was also significantly elevated on day 1 and remained such. Collectively, these data suggest that both pro- and anti-inflammatory responses were triggered after laparoscopic surgery in CRC.

### Association of Perioperative Immune Responses With Patients’ Clinicopathological Features

Perioperative fluctuations of several T cell and NK cell subsets were associated with tumor locations, but not B cells, monocytes, and DCs (**Supplementary Figures 7B, 8-10**). The frequencies of a CD4(+) effector (T9) and a CD8(+) effector (T18) cluster were significantly higher in patients with colon tumor than patients with rectal tumor on days 1 and 3 after surgery (**Supplementary Figure 8**). Patients with colon cancer tend to have significantly higher frequencies of NK6 and NK15 (intermediate), NK10 (immature), and NK16 (mature) subsets than patients with rectal cancer after surgery (**Supplementary Figure 10**).

The perioperative alterations of several monocyte clusters were affected by tumor stage, whereas T cells, B cells, DCs, and NK cells were not (**Supplementary Figures 7C, 11-13**). The frequencies of classical monocyte subsets MD6, MD14, and MD16 were significantly higher in patients with stage II CRC than patients with stage III disease after surgery (**Supplementary Figure 12**).

The frequencies of several NK subsets were associated with patients’ age, but not the other cell lineages (**Supplementary Figures 7D, 14–16**). Patients less than 60 years had significantly higher frequencies of intermediate NK cell subsets (NK6 and NK12), and significantly less mature (NK11) and immature (NK13) NK cells than patient older than or equal to 60 years, post-operatively (**Supplementary Figure 16**).

Smoking impacted perioperative frequencies of several T cell subsets (**Supplementary Figure 17**). Non-smokers tend to have significantly higher frequencies of CD8(+) naïve T cells (T7) and CD4(+) effector cells (T21), and less CD8(+) effector cells (T14) than smokers. The perioperative proportions of the other immune cells were not associated with smoking (**Supplementary 7E, 18–19**).

## Discussion

Single-cell mass cytometry allows for meticulous partitioning of perioperative immune cells in patients who undergo laparoscopic surgical resection of CRC. Simultaneous monitoring of major immune lineages provided an overview of surgery-caused alterations across the immune system, including cell augments and contractions and precisely timed changes in immune cell distribution in both innate and adaptive compartments, providing an avenue for improving clinical outcomes (33).

The depression of cellular immunity is associated with surgical stress in locoregional CRC. Indeed, major abdominal operations have long been associated with post-operative immunosuppression (49). Surgery-attenuated immune function is reflected by a marked reduction in total systemic CD4(+) and CD8(+) T cell counts (50). Similarly, our results showed that the frequency of total T cell was significantly lower on post-operative day 1 comparing to the baseline. A possible mechanism is the significantly increased apoptosis of CD4(+) and CD8(+) T cells after surgery (51). Moreover, we showed that myeloid-derived suppressor cell (MDSC)-like CD11b(+)CD33(+)CD14(+)CD16(−) monocytes with low HLA-DR expression levels were markedly elevated immediately after surgery comparing to the pre-surgical level. Low HLA-DR expression in circulating monocytes is a signature of immunoparalysis (35). Among the mediators produced during inflammation, cortisol (52) and IL-10 (53) have been shown to contribute to the downregulation of HLA-DR in CD14(+) cells. Post-surgical IL-10 levels were significantly higher than baseline in CRC (**Figure 6**). Internalization of the HLA-DR β chain after ubiquitination by MATCH ubiquitin ligase is another possible mechanism of HLA-DR downregulation (54). Furthermore, we show that the frequency of post-operative NK cells were decreased, as described elsewhere (22, 47). Surgical stress induces metabolic changes, leading to increased secretion of catecholamines, glucocorticoids, and prostaglandins. Each of these substances has been linked to the toxic impairment of NK cells (55–57).

Previous studies suggest that laparoscopy-assisted surgery allows for a rapid return to the pre-operative status (22). A plausible explanation is that the systemic immune response is better preserved, and local inflammation is less pronounced after laparoscopy than after open surgery because the surgical trauma is limited (22, 58). As expected, when comparing the proportions of each immune cell lineage, our results show that laparoscopic surgery-induced immune perturbations of total T cells, monocytes, and NK cells were restored to pre-surgical levels within seven days after surgery. Strikingly however, even the surgical trauma is limited, single-cell analysis shows that laparoscopic surgery-induced immune disorders are complex and prolonged, and the dynamics of single-cell clusters are different from those of the bulk population. Although the frequency of total T cells recovered to the pre-surgical level, the majority of the single clusters that were significantly affected did not recover until post-operative day 7 (**Figure 3**). The most remarkably influenced NK cell clusters did not return to baseline levels after 7 days of surgery. Furthermore, HLA-DR expression in both the bulk classical monocyte population and single monocyte clusters did not return to the pre-surgical level. Except for altered cell phenotypes and frequencies, pro- and anti-inflammatory cytokines remained high after surgery. Collectively, single-cell analysis indicated that surgical stress was profound and perioperative immune perturbation was far from recovery within 7 days after surgery. Consistently, a genome-wide analysis of pooled circulating leukocytes showed that up to 80% of the leukocyte transcriptome is altered after surgery and is prolonged for days and weeks (59).

Moreover, our results suggest that resection surgery for CRC differentially affects immune cell compartments. Naïve, effector, and memory T cells; classical and intermediate monocytes; mDCs and pDCs; and immature and mature NK cells showed distinct fluctuation patterns upon surgical stress. We observed inconsistent fluctuations in clusters within the same cell compartment. In addition, the expression levels of single markers in the same cell compartments were different. The differential modulation of HLA-DR in monocyte subpopulations likely results from different exogenous signals (60).

Increasing evidence support that immunological control of cancer can be impacted by tumor resection (61, 62). Our results suggest that patients with colon cancer had higher frequencies of some effector T cell subsets and NK cell subsets than patients with rectal cancer. It has been reported that patients with rectal cancer have a higher incidence to suffer lung metastasis after surgery than patients with colon cancer (63, 64). High percentages of post-operative peripheral CD3(+), CD4(+), and CD8(+) T cells predict lower recurrence free survival in patients with CRC as they exert anti-tumor functions (65). The post-operative environment suppresses NK cells, which are critical mediators in the formation of metastases immediately following surgery (66). Moreover, it has been shown that absolute monocyte count decrease predicts worse overall survival and disease-free survival in esophageal squamous cell carcinoma undergoing curative resection (67). In our study, both elderly and patient with stage III tumors showed lower frequencies of certain intermediate NK cell subsets (NK6, for instance) which has been associated with post-operative anti-tumor immunity (44). Patients with stage II CRCs showed significantly higher frequencies of several monocyte subsets than patients with stage III diseases. Consistently, the mortality of older patients is much higher than younger patients, and patients diagnosed with late-stage tumors have worse prognosis than patients diagnosed with early-stage tumors (1). Moreover, our results indicate smokers had lower frequencies in naïve and certain effector T cell subsets. Cigarette smoking has been associated with higher all-cause and CRC-specific mortality among patients with locoregional CRC (68). Previous studies tested bulk immune cell lineages and overlooked the influences of cell subpopulations on cancer prognosis. Single-cell mass cytometry provides advanced monitoring of perioperative immune responses by simultaneously comparing the levels of multiple cell lineages and their subsets. Future studies are required to elucidate the correlation between fluctuation of single-cell subsets with clinical outcomes.

The perioperative period provides an opportunity for cancer cells to metastasize and a therapeutic window in which the metastatic process can be intervened (9–11). For example, our study showed a dramatic decrease of HLA-DR expression on monocytes after surgery, reversing of which can possibly enhance antigen-presenting of monocytes (34). In a recent study, nanoparticles were used to deliver antigenic material to antigen-presenting cells, which result in increased expression of costimulatory molecules CD80, CD83, CD86, and HLA-DR (69). Moreover, the decrease in total frequencies of circulating T cells and NK cells hint at peri-operative immune suppression. Immune stimulatory agents could be used to reverse systemic immune changes in the context of surgical excision of tumors. For instance, Toll-like receptor (TLR) agonists play a vital role in activating both innate and adaptive immunity. Upon TLR-4 activation, APCs generate vast amounts of proinflammatory cytokines, including INF-α, which in turn stimulate the proliferation of CD4(+) T cells (70). TLR-4 engagement also induces the production of IFN-γ, TNF-α, perforin, and granzyme B by CD8(+) cytotoxic T cells and NK cells (71). It has been shown that a synthetic TLR-4 agonist, Glucopyranosyl Lipid A (GLA)-SE, provides a promising effect on cancer progression in the perioperative context in animal models (72).

There are certain limitations for this study. Firstly, the signature of leucocytes in peripheral blood may be affected by a variety of confounding factors, including age, race, physical fitness, obesity, autoimmunity, post-operative infection, exposure to viral antigens, the magnitude of surgical stress, and so on. To minimize these effects, we enrolled Chinese patients without post-operative infections, without comorbidities, without immune-related diseases, and underwent similar surgical procedures, which enabled the identification of key immune alterations from CRC radical surgery. Experimental and clinical evidence suggests that because of limited surgical trauma and improved preservation of immune functions, the minimally invasive laparoscopic approach is usually associated with clinically relevant benefits during the first weeks after surgery (22, 73). We expected to determine the baseline immune correlates for laparoscopic CRC resection surgery, since currently more than 95% of radical surgeries for locoregional CRC are performed by laparoscopy in our hospital, and laparoscopic surgery is the inevitable trend of surgical development. Our observations in alterations of total T cells, monocytes, DCs, and NK cells are in agreement with other studies about immunological effects of CRC surgeries. However, further study is needed to elucidate whether the changes seen are directly oncologic relevant and specific for CRC surgery or are generic to all abdominal surgeries. The sample size is relatively small, and the numbers of female and male patients were unbalanced, future studies with larger cohorts are needed to validate and generalize these findings. Additionally, longitudinal studies are needed to determine the correlation between perioperative immune alterations of different cell subsets with their impact on the biology and outcomes of CRC.

## Data Availability Statement

The raw data supporting the conclusions of this article will be made available by the authors, without undue reservation.

## Ethics Statement

The studies involving human participants were reviewed and approved by Research Ethics Committee of Peking University Cancer Hospital & Institute, Beijing, China (No. 2019KT33). The patients/participants provided their written informed consent to participate in this study.

## Author Contributions

CZ collected and processed clinical samples. JD, CZ, and XS designed the study. JD and CZ drafted the manuscript. JD and CZ analyzed and interpreted the data. ZW and BJ helped with administrative issues and data interpretation. XS reviewed and finalized the manuscript. All authors contributed to the article and approved the submitted version.

## Funding

This work was supported by the National Natural Science Foundation of China (No. 81972678) and the Beijing Natural Science Foundation (No. 7192037) to JD, the National Natural Science Foundation of China (No. 81672439, 81872022, and 82171720) and the Beijing Natural Science Foundation (No. 7162039) to XS, the National Natural Science Foundation of China (No. 82073357) to ZW.

## Conflict of Interest

The authors declare that the research was conducted in the absence of any commercial or financial relationships that could be construed as a potential conflict of interest.

## Publisher’s Note

All claims expressed in this article are solely those of the authors and do not necessarily represent those of their affiliated organizations, or those of the publisher, the editors and the reviewers. Any product that may be evaluated in this article, or claim that may be made by its manufacturer, is not guaranteed or endorsed by the publisher.
