# Supplementary material for: Monitoring Pre- and Post-Operative Immune Alterations in Patients With Locoregional Colorectal Cancer Who Underwent Laparoscopy by Single-Cell Mass Cytometry
Source: Front Immunol. 2022 Feb 3;13:807539. doi: 10.3389/fimmu.2022.807539 (PMC8850468; doi:10.3389/fimmu.2022.807539)
Supplement: Supplementary file 2 [file Table_1.pdf]

Supplementary Table 1. Clinicopathological characteristics of patients with CRC

ID	Gender	Age	TNM	Stage	Location	Size (cm)	Histology	Differentiation	Lymph node invasion	Vascular thrombus	Smoking
376	Male	57	pT4aN1bM0	IIIB	Rectum	4.1 x4x1	Adenocarcinoma	Moderate	Yes	No	Yes
378	Male	72	pT4bN1aM0	IIIC	Sigmoid colon	3.5x3x0.5	Adenocarcinoma	Moderate	Yes	Yes	Yes
382	Male	44	pT3N1aM0	IIIB	Rectum	4x3.5x0.7	Adenocarcinoma	Moderate-poor	Yes	No	No
386	Male	69	pT2N0M0	I	Right-sided colon	2x1.5x0.5	Adenocarcinoma	Moderate	No	No	Yes
389	Female	65	pT3N0M0	IIA	Left-sided colon	2.5x2x1	Adenocarcinoma	Moderate	No	No	No
390	Male	55	pT3N0M0	IIA	Rectum	5x5x1.1	Adenocarcinoma	Moderate	No	No	Yes
391	Male	50	pT4aN1bM0	IIIB	Rectum	6x5x2.2	Adenocarcinoma	Moderate	Yes	Yes	Yes
392	Female	52	pT3N1bM0	IIIB	Right-sided colon	3.2x2.5	Adenocarcinoma	Moderate	Yes	Yes	No
393	Male	60	pT4aN2bM0	IIIC	Sigmoid colon	7x3.5	Adenocarcinoma	Moderate-poor	Yes	Yes	Yes
394	Male	64	pT3N0M1	IIA	Sigmoid colon	4x3	Adenocarcinoma	Moderate	No	No	Yes
395	Male	61	pT3N0M0	IIA	Sigmoid colon	3x2x0.8	Adenocarcinoma	Moderate	No	No	No
396	Female	49	pT4aN1bM0	IIIB	Right-sided colon	5.5x4x1.2	Adenocarcinoma	Moderate	Yes	No	No
397	Male	62	pT3N1bM0	IIIB	Rectum	2.3x2.1x0.9	Adenocarcinoma	Moderate	Yes	Yes	Yes
398	Female	38	pT3N0M0	IIA	Right-sided colon	8x5x1.5	Adenocarcinoma	Moderate	No	No	No
399	Male	62	pT3N0M0	IIA	Sigmoid colon	2x2x0.6	Adenocarcinoma	Moderate	No	No	Yes

400	Male	54	pT3N0M0	IIA	Rectum	6x4x0.8	Adenocarcinoma	Moderate	No	No	Yes
401	Male	51	pT3N1aM0	IIIB	Right-sided colon	6x3.5x1	Mucinous carcinoma		Yes	No	Yes
402	Female	52	pT3N1bM0	IIIB	Sigmoid colon	3.5x2.8x0.9	Adenocarcinoma	Moderate	Yes	Yes	No
403	Male	69	pT4bN0M0	IIC	Rectum	6.5x3.5x1.5	Adenocarcinoma	Moderate	No	No	Yes
404	Male	55	pT3N0M0	IIA	Right-sided colon	8x3	Adenocarcinoma	Moderate-poor	No	No	Yes
405	Male	50	pT3N0M0	IIA	Right-sided colon	6.5x5x2	Adenocarcinoma	Moderate	No	No	Yes
406	Female	70	pT3N0M0	IIA	Right-sided colon	6x5.5x1.5	Adenocarcinoma	Moderate	No	No	No
407	Female	41	pT2N0M0	I	Sigmoid colon	6x4x2	Adenocarcinoma	Moderate	No	No	No
408	Male	56	pT4aN1aM0	IIIB	Right-sided colon	8x7x2	Adenocarcinoma	Moderate-poor	Yes	Yes	No
