# Supplementary material for: Monitoring Pre- and Post-Operative Immune Alterations in Patients With Locoregional Colorectal Cancer Who Underwent Laparoscopy by Single-Cell Mass Cytometry
Source: Front Immunol. 2022 Feb 3;13:807539. doi: 10.3389/fimmu.2022.807539 (PMC8850468; doi:10.3389/fimmu.2022.807539)
Supplement: Supplementary file 3 [file Table_2.docx]

**Supplementary Table 2. Mass-tagged antibody panel**

| **Isotope** | **Antibody** | **Clone** | **Catalog** | **Source** |
| --- | --- | --- | --- | --- |
| 106Cd  112Cd  114Cd  163Dy | Purified anti-human CD45 (Maxpar® Ready) | HI30 | 304045 | Biolegend |
| 111Cd | Purified anti-human CD4 (Maxpar Ready) | RPA-T4 | 300541 | Biolegend |
| 113Cd | Purified anti-human CD8a (Maxpar® Ready) | RPA-T8 | 301053 | Biolegend |
| 116Cd | Purified anti-human CD3 (Maxpar® Ready) | UCHT1 | 300443 | Biolegend |
| 141Pr | Purified anti-human CD196 (CCR6) (Maxpar® Ready) | G034E3 | 353427 | Biolegend |
| 142Nd | Purified anti-human CD14 (Maxpar® Ready) | M5E2 | 301843 | Biolegend |
| 143Nd | Purified anti-human CD11c (Maxpar® Ready) | Bu15 | 337221 | Biolegend |
| 144Nd | Purified anti-human CD45RO (Maxpar® Ready) | UCHL1 | 304239 | Biolegend |
| 145Nd | Purified anti-human CD16 (Maxpar® Ready) | 3G8 | 302051 | Biolegend |
| 146Nd | Purified anti-human CD45RA (Maxpar® Ready) | HI100 | 304143 | Biolegend |
| 147Sm | Purified anti-human CD183 (CXCR3) (Maxpar® Ready) | G025H7 | 353733 | Biolegend |
| 148Sm | Purified anti-human CD95 (Fas) (Maxpar® Ready) | DX2 | 305631 | Biolegend |
| 149Sm | Purified anti-human CD161 (Maxpar® Ready) | HP-3G10 | 339919 | Biolegend |
| 150Sm | Purified anti-human CD28 (Maxpar® Ready) | CD28.2 | 302937 | Biolegend |
| 151Eu | Purified anti-human CD123 (Maxpar® Ready) | 6H6 | 306027 | Biolegend |
| 152Sm | Purified anti-human CD127 (MaxPar® Ready) | A019D5 | 351337 | Biolegend |
| 153Eu | Purified anti-human CD1c | L161 | 331502 | Biolegend |
| 154Gd | Purified anti-human CD185 (CXCR5) (Maxpar® Ready) | J252D4 | 356902 | Biolegend |
| 155Gd | Purified anti-human CD279 (PD-1) (Maxpar® Ready) | EH12.2H7 | 329941 | Biolegend |
| 156Gd | Purified anti-human CD194 (CCR4) | L291H4 | 359402 | Biolegend |
| 158Gd | Purified anti-human CD33 (Maxpar® Ready) | WM53 | 303419 | Biolegend |
| 159Tb | Purified anti-human CD15 (Maxpar® Ready) | W6D3 | 323035 | Biolegend |
| 160Gd | Purified anti-human CD195 (CCR5) | J418F1 | 359102 | Biolegend |
| 161Dy | Purified anti-human CD66b | G10F5 | 305102 | Biolegend |
| 162Dy | Purified anti-human CTLA-4 | L3D10 | 349902 | Biolegend |
| 164Dy | Purified anti-human CD11b (Maxpar® Ready) | ICRF44 | 301337 | Biolegend |
| 165Ho | Purified anti-human CD27 (Maxpar® Ready) | O323 | 302839 | Biolegend |
| 166Er | Purified anti-human CD38 | HIT2 | 303502 | Biolegend |
| 167Er | Purified anti-human CD303 (BDCA-2) (Maxpar® Ready) | 201A | 354215 | Biolegend |
| 168Er | Purified anti-human CD56 (NCAM) (Maxpar® Ready) | HCD56 | 318345 | Biolegend |
| 169Tm | Purified anti-human CD25 | BC96 | 302602 | Biolegend |
| 170Er | Purified anti-human/mouse/rat CD278 (ICOS) | C398.4A | 313502 | Biolegend |
| 171Yb | Purified anti-human CD57 | HNK-1 | 359602 | Biolegend |
| 173Yb | Purified anti-human CD141 | M80 | 344102 | Biolegend |
| 174Yb | Purified anti-human HLA-DR (Maxpar® Ready) | L243 | 307651 | Biolegend |
| 175Lu | Purified anti-human CD19 (Maxpar® Ready) | HIB19 | 302247 | Biolegend |
| 176Yb | Purified anti-human CCR7 (MaxPar® Ready) | G043H7 | 353237 | Biolegend |
|  | Maxpar® X8 Multimetal Labeling Kit—40 Run |  | 201300 | Fluidigm |
|  | Cell-ID™ IdU |  | 201127 | Fluidigm |
|  | Cell-ID™ Intercalator-Ir |  | 201192A | Fluidigm |
