# Supplementary material for: Monitoring Pre- and Post-Operative Immune Alterations in Patients With Locoregional Colorectal Cancer Who Underwent Laparoscopy by Single-Cell Mass Cytometry
Source: Front Immunol. 2022 Feb 3;13:807539. doi: 10.3389/fimmu.2022.807539 (PMC8850468; doi:10.3389/fimmu.2022.807539)

Supplementary figure legend

Supplementary Figure 1. Cell counts of each sample that passed pre-processing. The non-beads, single, live, and debarcoded CD45(+) cell count from each sample that passed pre-processing are shown.

Supplementary Figure 2. Gating strategy showing by a representative patient. PBMCs were labeled with the indicated set of antibodies (Supplementary Table 2), examined by mass cytometry, and then analyzed by Cytobank. Cells were pre-gated as non-beads, single, live, and debarcoded (by CD45 labeled with different metal isotopes). The major immune cell lineages and cell subsets were identified through the indicated series of gating steps. mDC, myeloid dendritic cell; pDC, plasmacytoid dendritic cell; NK, natural killer.

Supplementary Figure 3. Characterization of immune cells in CRC use mass cytometry, related to Figure 1.

(A) The composition of the average frequencies of major immune lineages in the CD45+ compartment of each time point is shown, supplementing Figure 1F and 1G. (B) FIt-SNE of immune cells colored by PhenoGraph clusters at different time points.

Supplementary Figure 4. Frequencies of immune cell clusters for each sample.

Dendrogram showing data hierarchically clustering. Clusters are indicated with colors. Sample IDs are listed on the left.

Supplementary Figure 5. In-depth analysis of perioperative monocyte and DC, related to Figure 4.

(A) FIt-SNE plots of normalized marker expression for 10,000 cells randomly selected from all patients. (B) Cluster-specific analysis of monocyte frequencies during the perioperative period. *p < 0.05, **p < 0.01, ***p < 0.001, ****p < 0.0001 by Krustal-Wallis analysis with post-hoc Dunn test. P-value adjusted with the Benjamini-Hochberg method.

Supplementary Figure 6. In-depth analysis of NK cells, related to Figure 5.

FIt-SNE plots of normalized marker expression for 10,000 cells randomly selected from all patients.

Supplementary Figure 7 Analysis of naïve and memory B cells.

(A) Boxplots showing the frequencies of the naïve and memory B cell clusters during the perioperative period (A) and at different tumor locations (B). *p < 0.05, **p < 0.01, ***p < 0.001, ****p < 0.0001 by Krustal-Wallis analysis with post-hoc Dunn test. P-value adjusted with the Benjamini-Hochberg method. (C-E) Boxplots comparing the perioperative B cell alterations in patients with stage II vs stage III tumors (C), in patients younger vs older than and equal to 60 years (D), and in smokers vs non-smokers (E), p values calculated by Wilcoxon test.

Supplementary Figure 8. Boxplots comparing the perioperative T cell alterations at different tumor locations. *p < 0.05, **p < 0.01, ***p < 0.001, ****p < 0.0001 by Krustal-Wallis analysis with post-hoc Dunn test. P-value adjusted with the Benjamini-Hochberg method.

Supplementary Figure 9. Boxplots comparing the perioperative Monocyte and DC

alterations at different tumor locations. (A) monocyte, (B) DC, *p < 0.05, **p < 0.01, ***p < 0.001, ****p < 0.0001 by Krustal-Wallis analysis with post-hoc Dunn test. P-value adjusted with the Benjamini-Hochberg method.

Supplementary Figure 10. Boxplots comparing the perioperative NK cell alterations at different tumor locations. *p < 0.05, **p < 0.01, ***p < 0.001, ****p < 0.0001 by Krustal-Wallis analysis with post-hoc Dunn test. P-value adjusted with the Benjamini-Hochberg method.

Supplementary Figure 11. Boxplots comparing the perioperative T cell alterations at different tumor stages. *p < 0.05, **p < 0.01, ***p < 0.001, ****p < 0.0001 by Wilcoxon test.

Supplementary Figure 12. Boxplots comparing the perioperative monocytes and DC alterations at different tumor stages. (A) monocyte, (B) DC, *p < 0.05, **p < 0.01, ***p < 0.001, ****p < 0.0001 by Wilcoxon test.

Supplementary Figure 13. Boxplots comparing the perioperative NK cell alterations at different tumor stages. *p < 0.05, **p < 0.01, ***p < 0.001, ****p < 0.0001 by Wilcoxon test.

Supplementary Figure 14. Boxplots comparing the perioperative T cell alterations in older and younger patients. *p < 0.05, **p < 0.01, ***p < 0.001, ****p < 0.0001 by Wilcoxon test.

Supplementary Figure 15. Boxplots comparing the perioperative monocyte and DC alterations in older and younger patients. (A) monocyte, (B) DC, *p < 0.05, **p < 0.01,

***p < 0.001, ****p < 0.0001 by Wilcoxon test.

Supplementary Figure 16. Boxplots comparing the perioperative NK cell alterations in older and younger patients. *p < 0.05, **p < 0.01, ***p < 0.001, ****p < 0.0001 by Wilcoxon test.

Supplementary Figure 17. Boxplots comparing the perioperative T cell alterations in patients with and without smoking habits. *p < 0.05, **p < 0.01, ***p < 0.001, ****p < 0.0001 by Wilcoxon test.

Supplementary Figure 18. Boxplots comparing the perioperative monocyte and DC alterations in patients with and without smoking habits. (A) monocyte, (B) DC, *p < 0.05, **p < 0.01, ***p < 0.001, ****p < 0.0001 by Wilcoxon test.

Supplementary Figure 19. Boxplots comparing the perioperative NK cell alterations in patients with and without smoking habits. *p < 0.05, **p < 0.01, ***p < 0.001, ****p < 0.0001 by Wilcoxon test.

Supplementary Figure 1

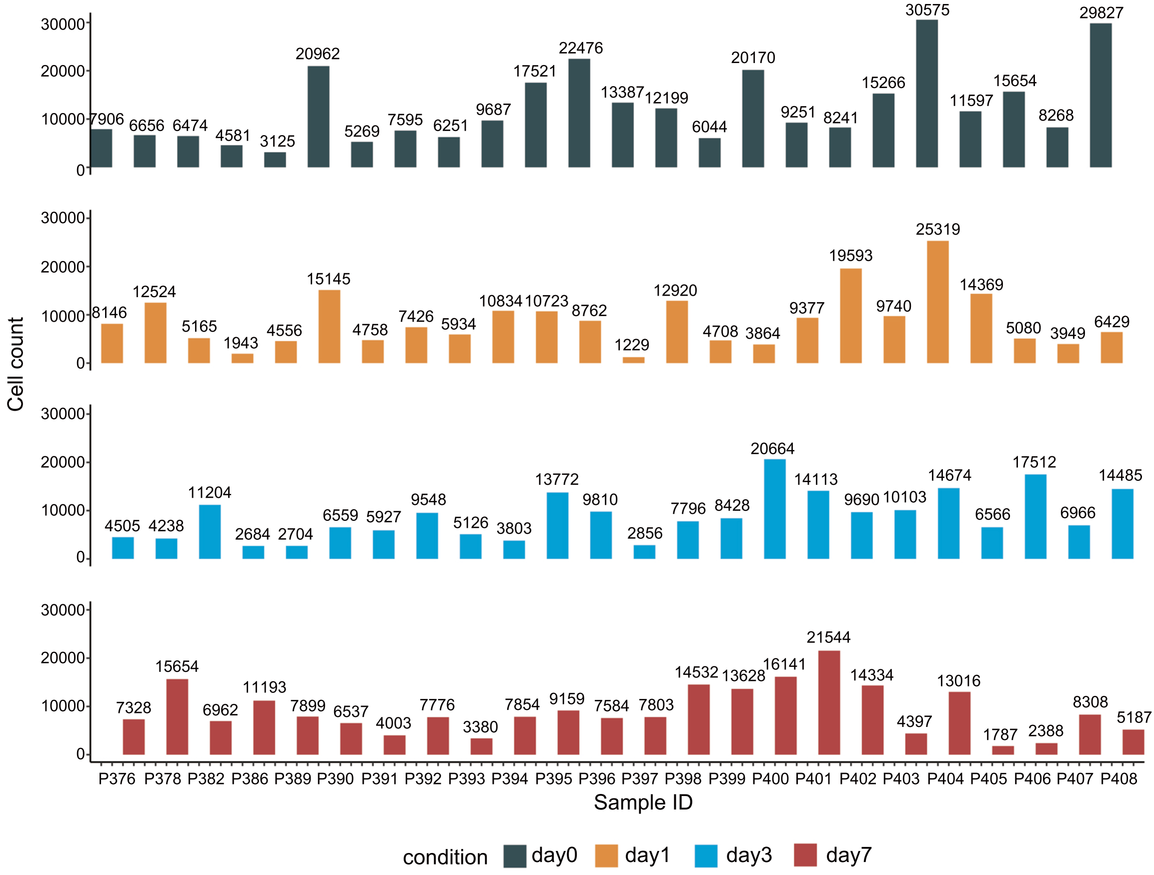

Supplementary Figure 2

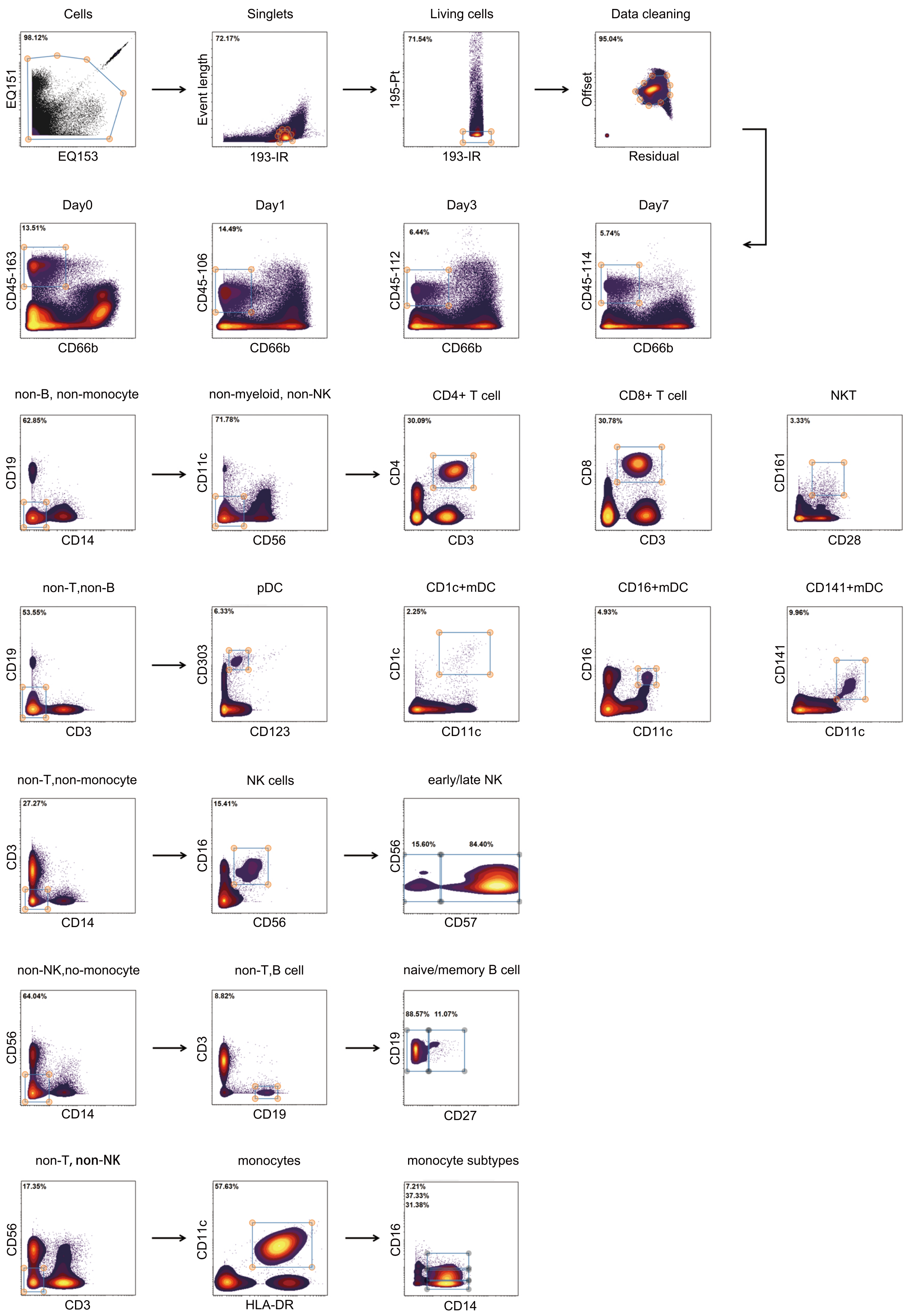

Supplementary Figure 3

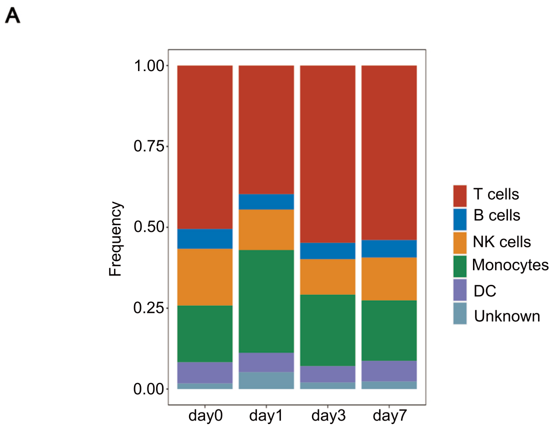

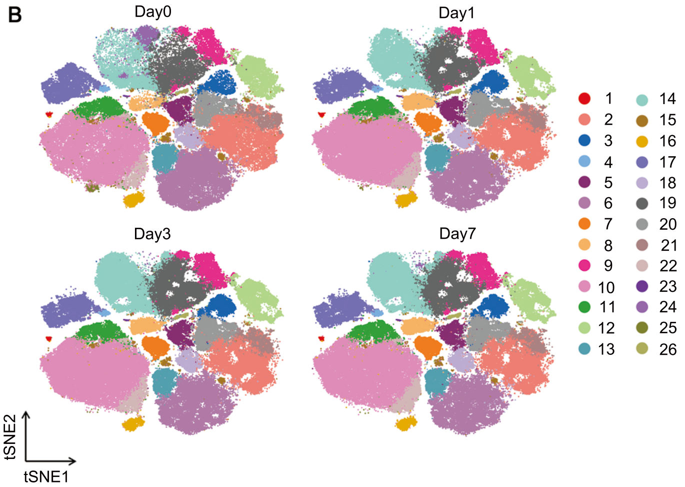

Supplementary Figure 4

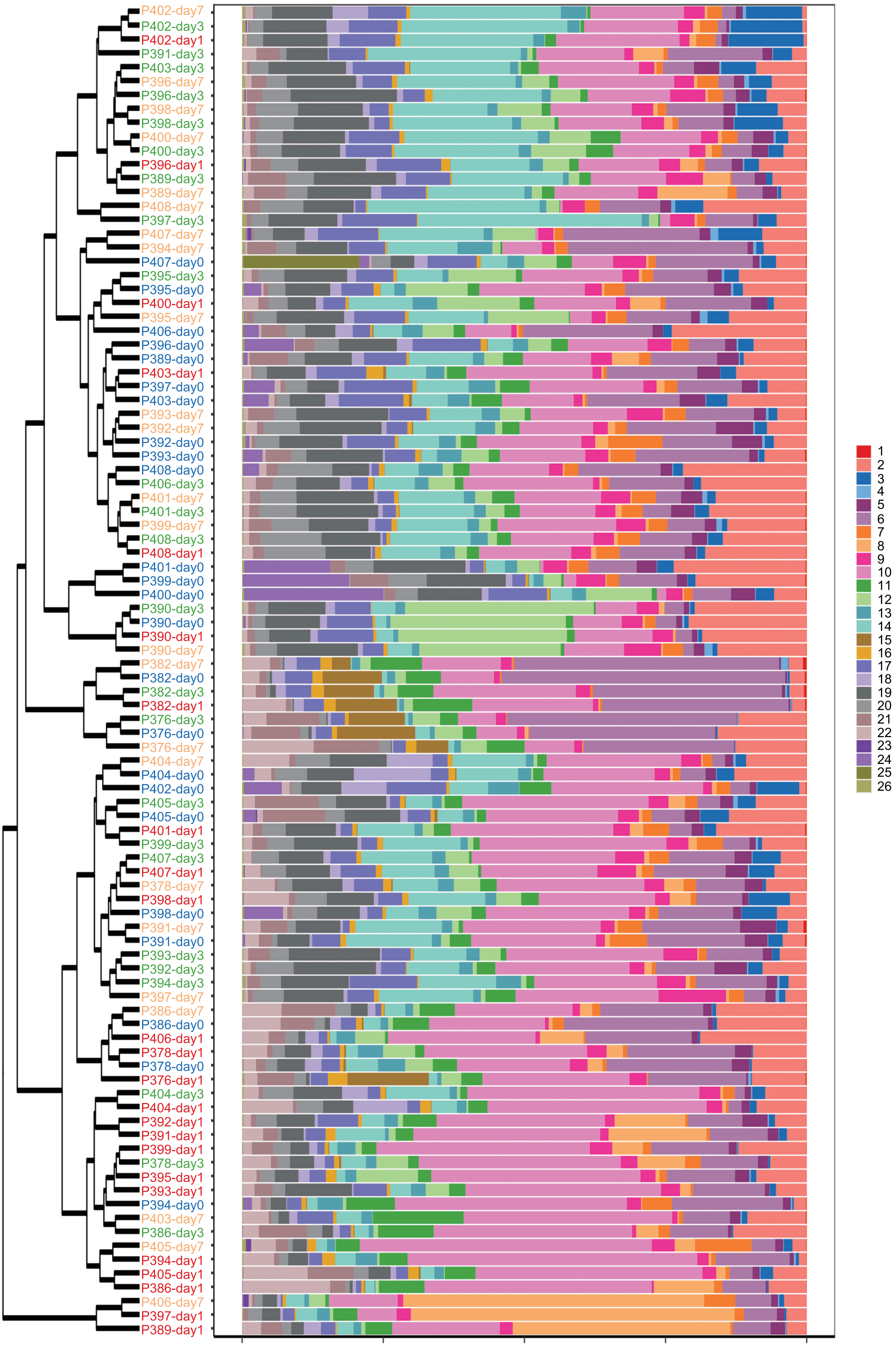

Supplementary Figure 5

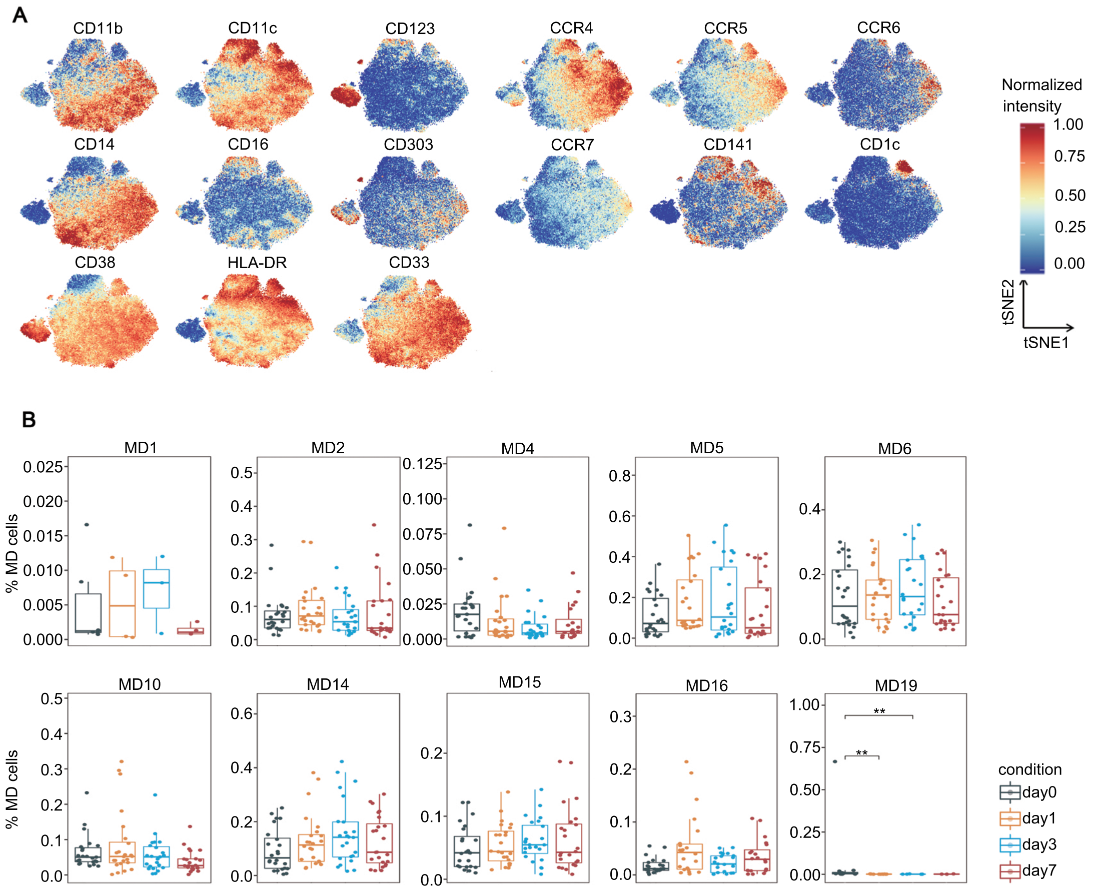

Supplementary Figure 6

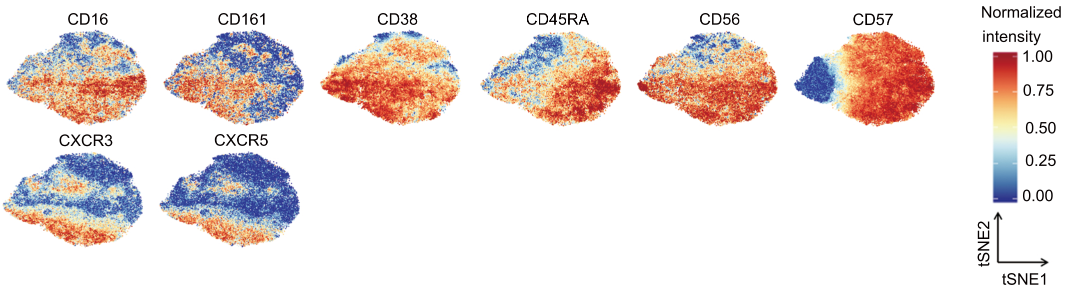

Supplementary Figure 7

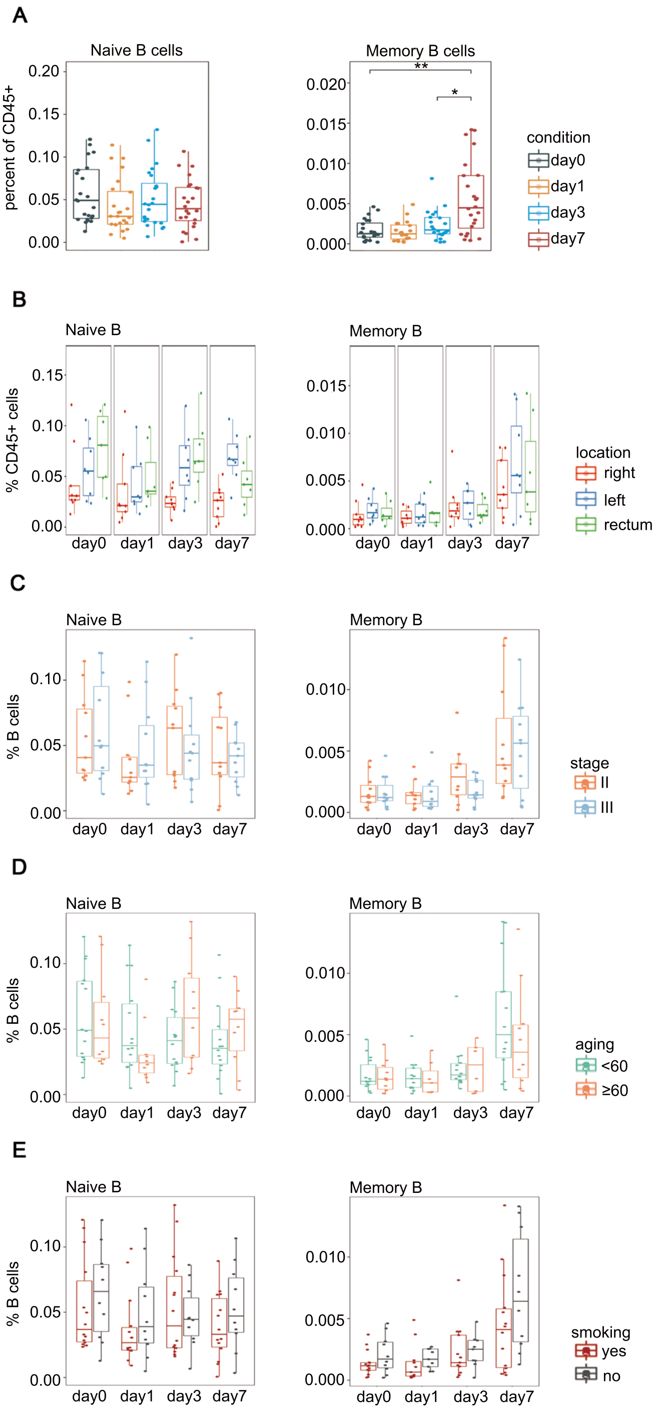

Supplementary Figure 8

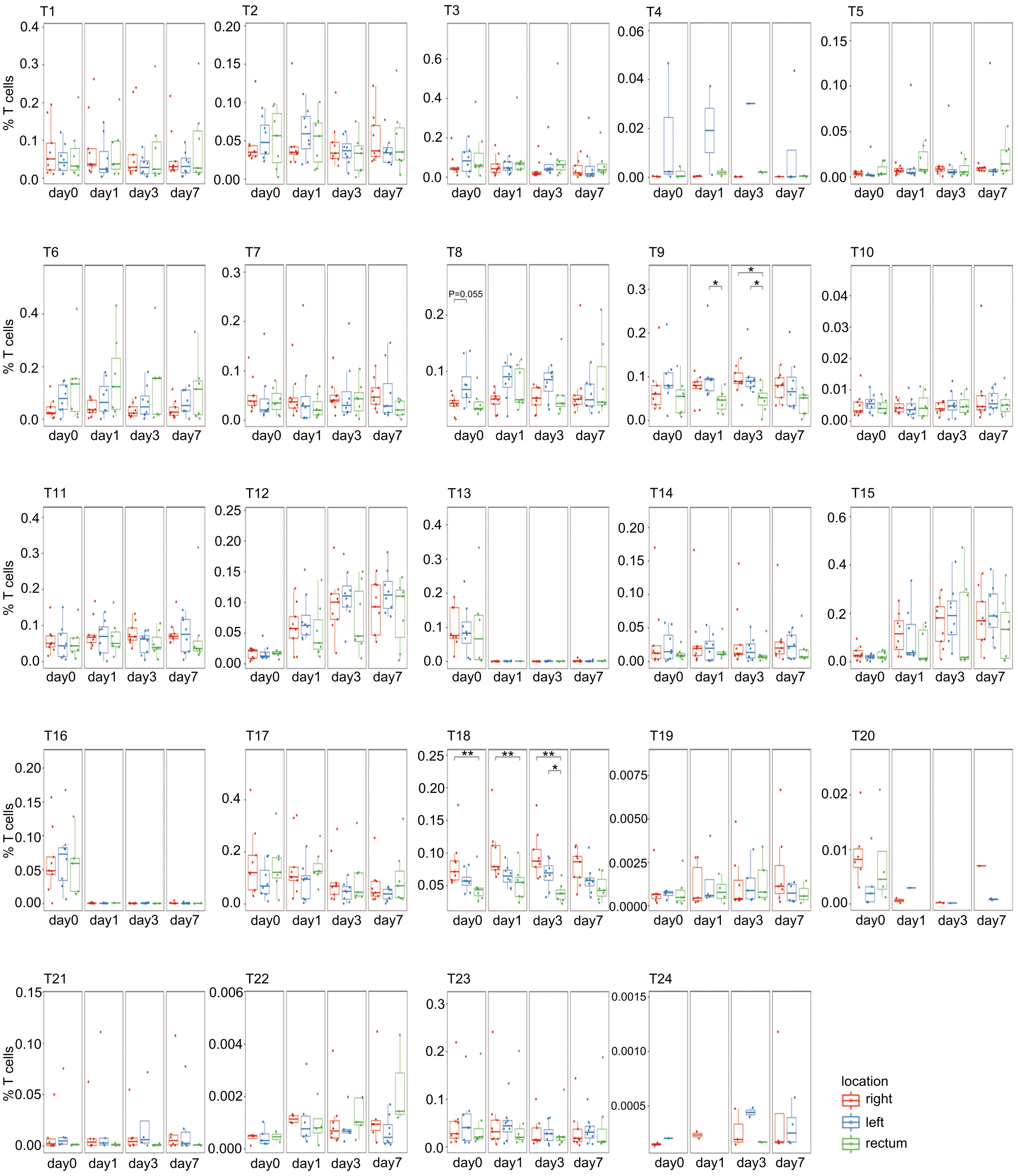

Supplementary Figure 9

A

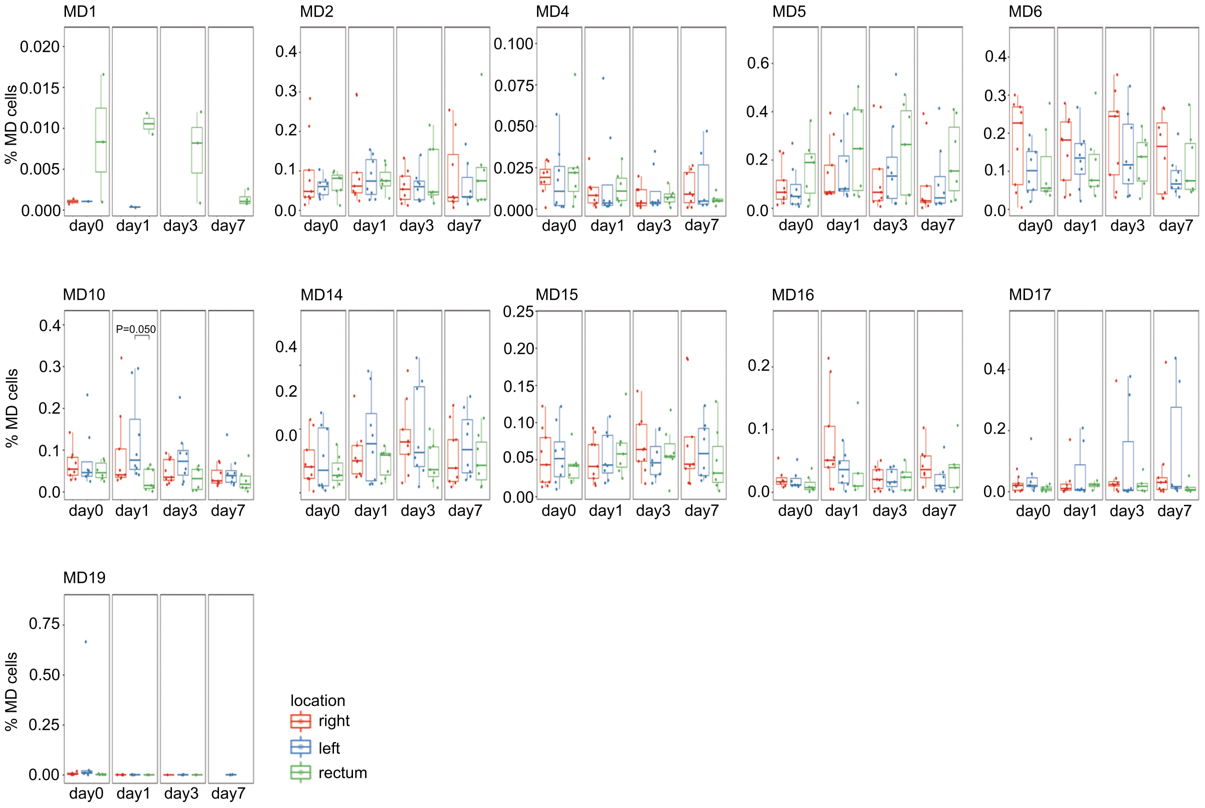

B

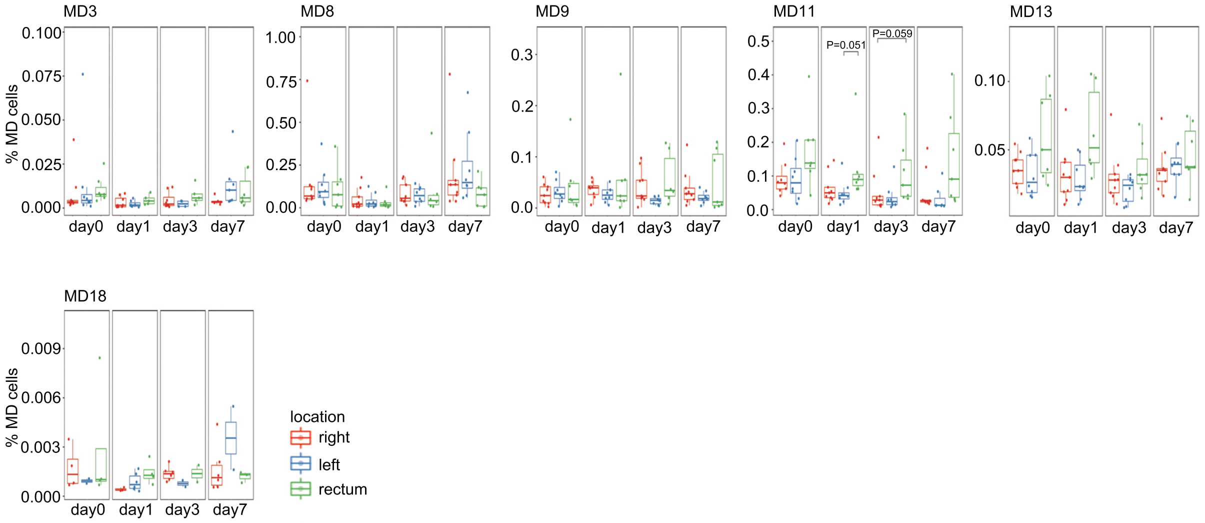

Supplementary Figure 10

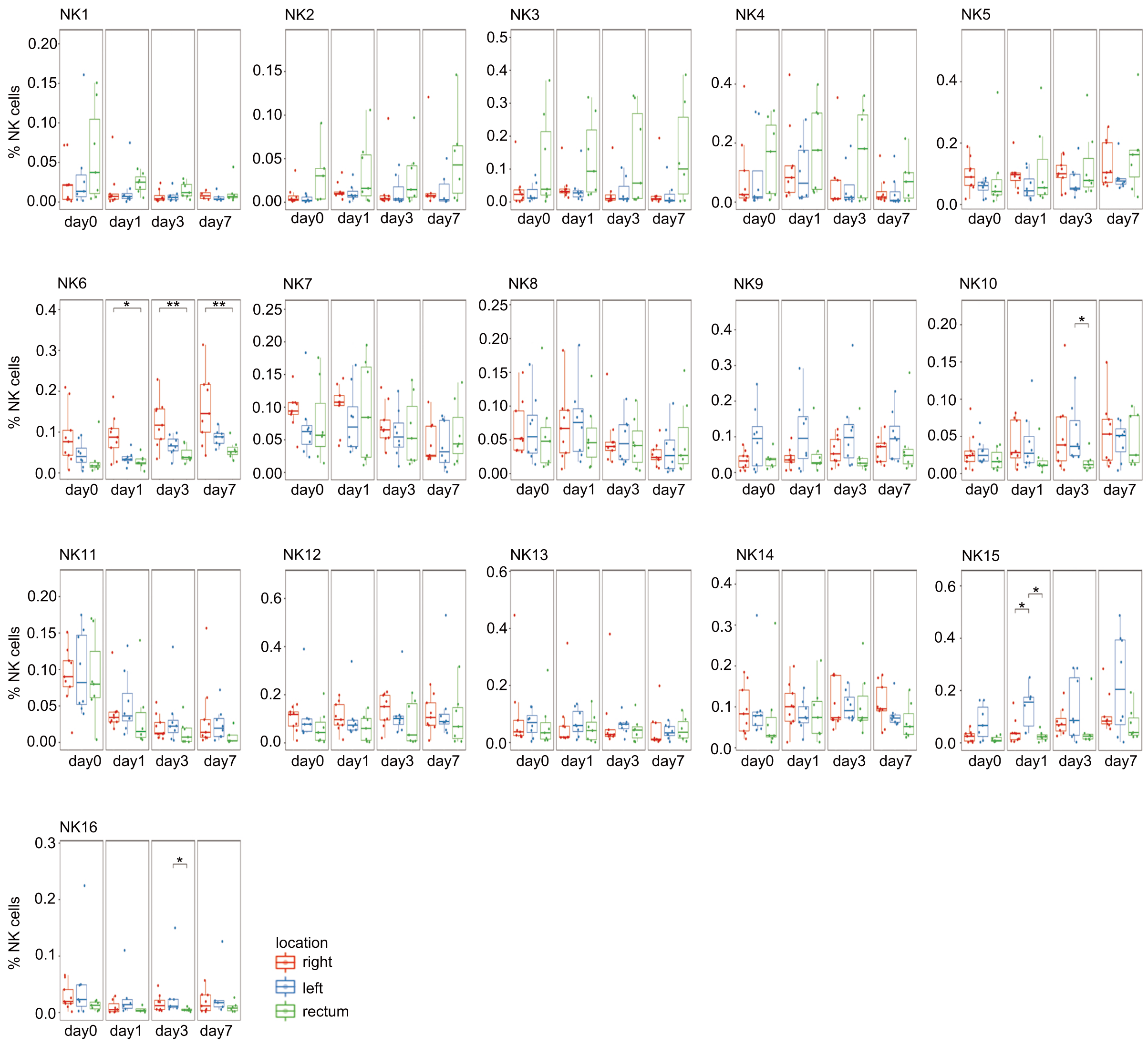

Supplementary Figure 11

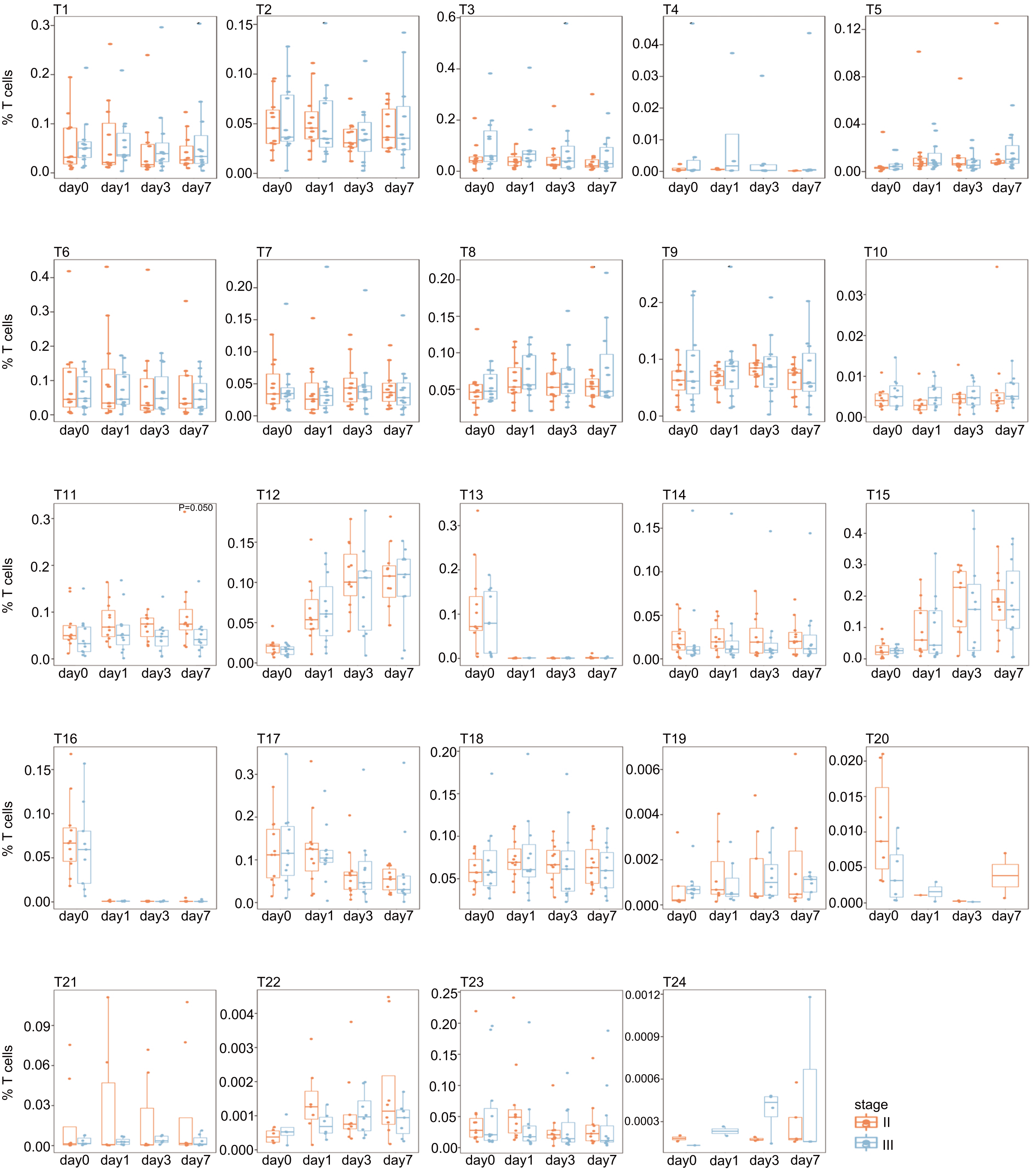

Supplementary Figure 12

A

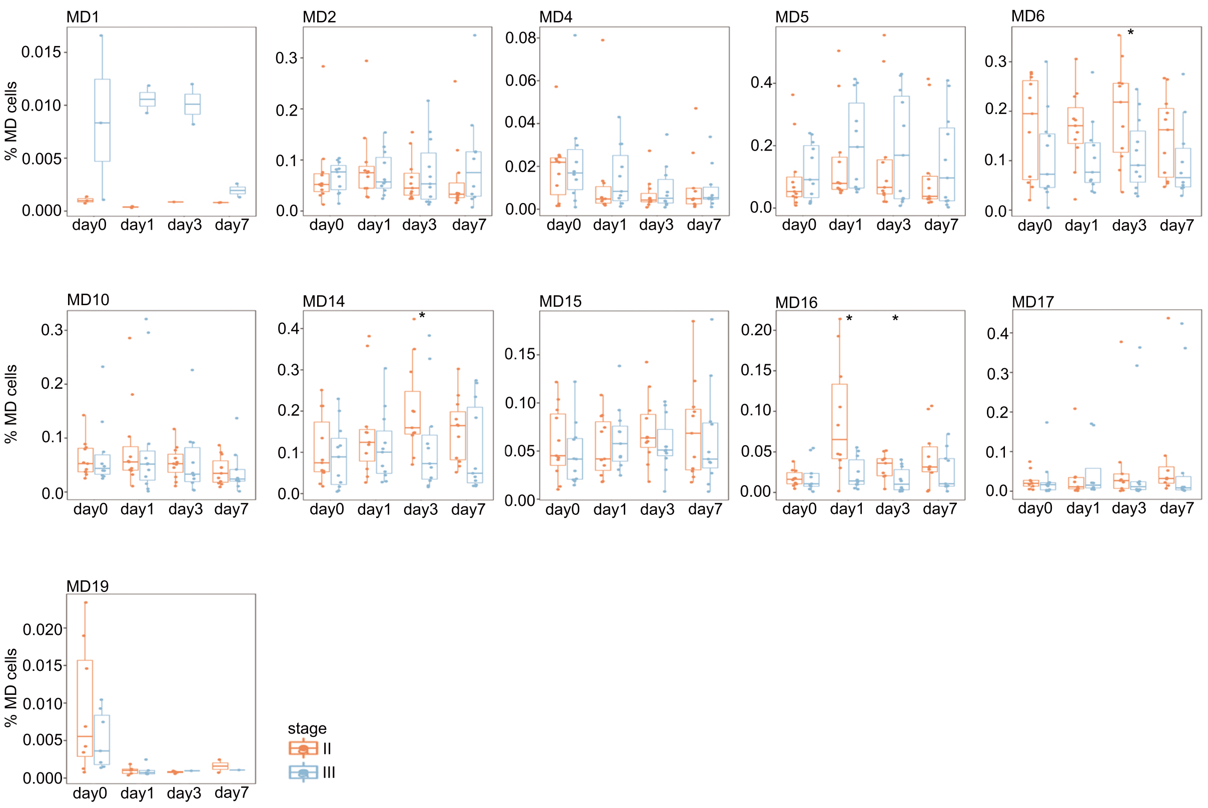

B

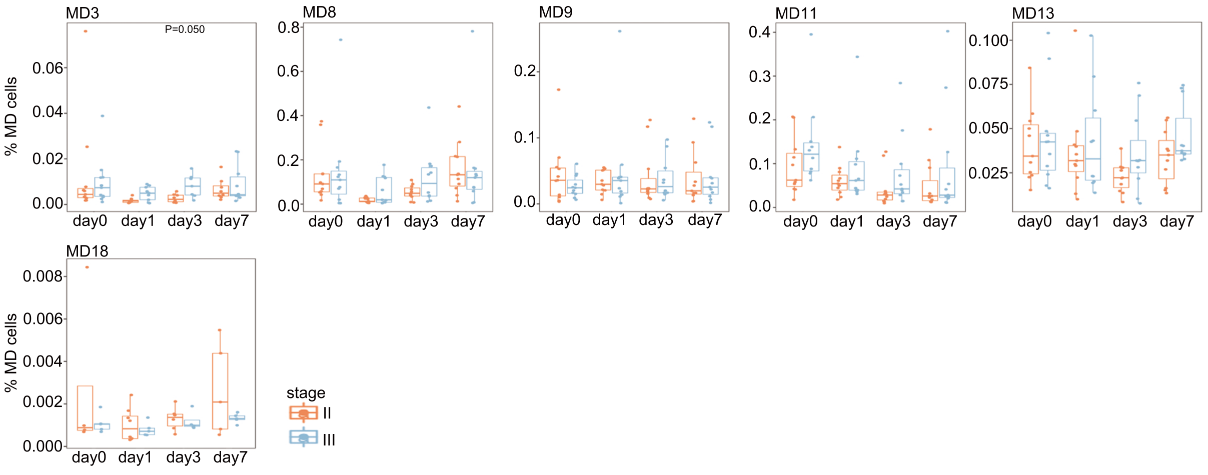

Supplementary Figure 13

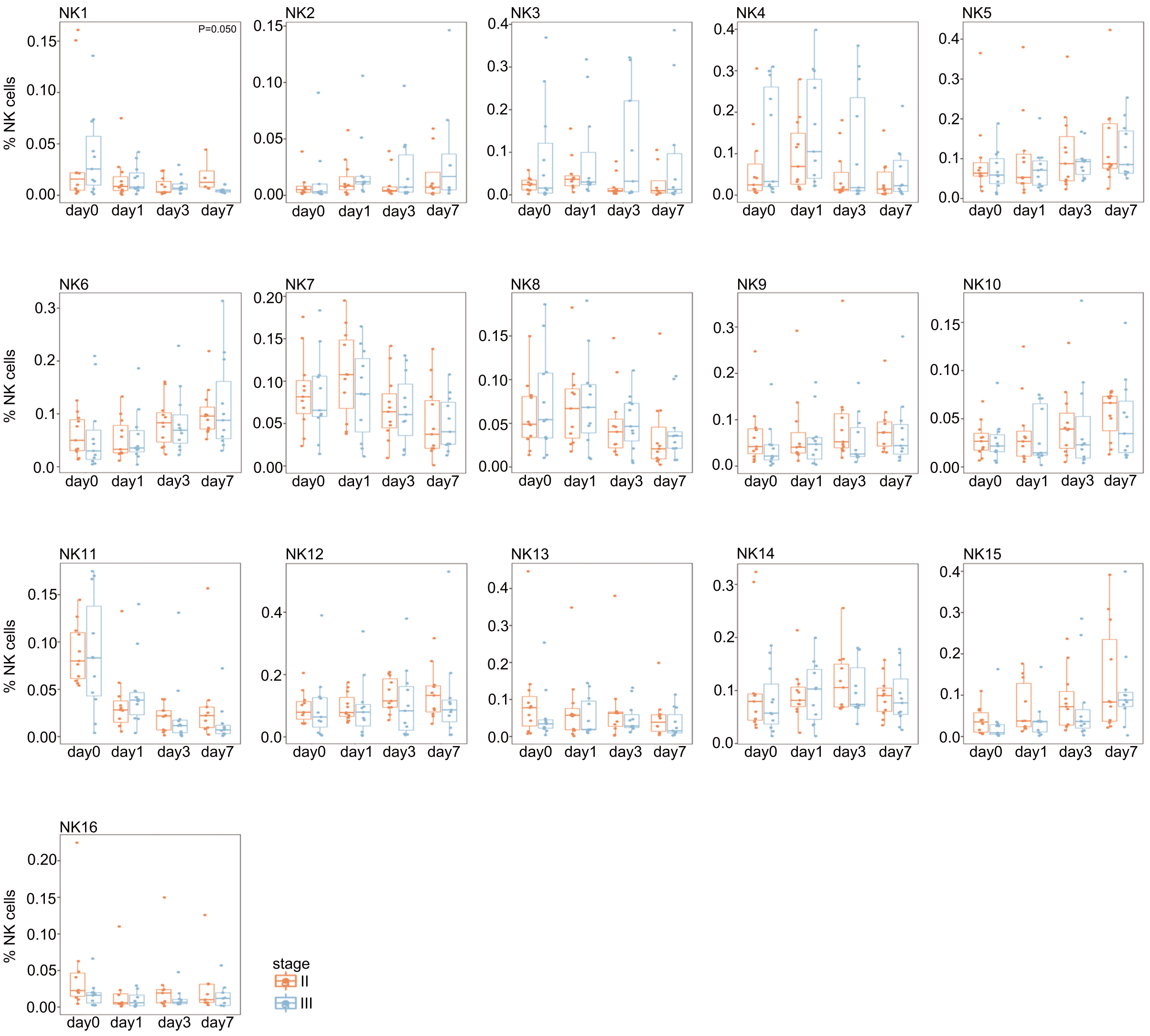

Supplementary Figure 14

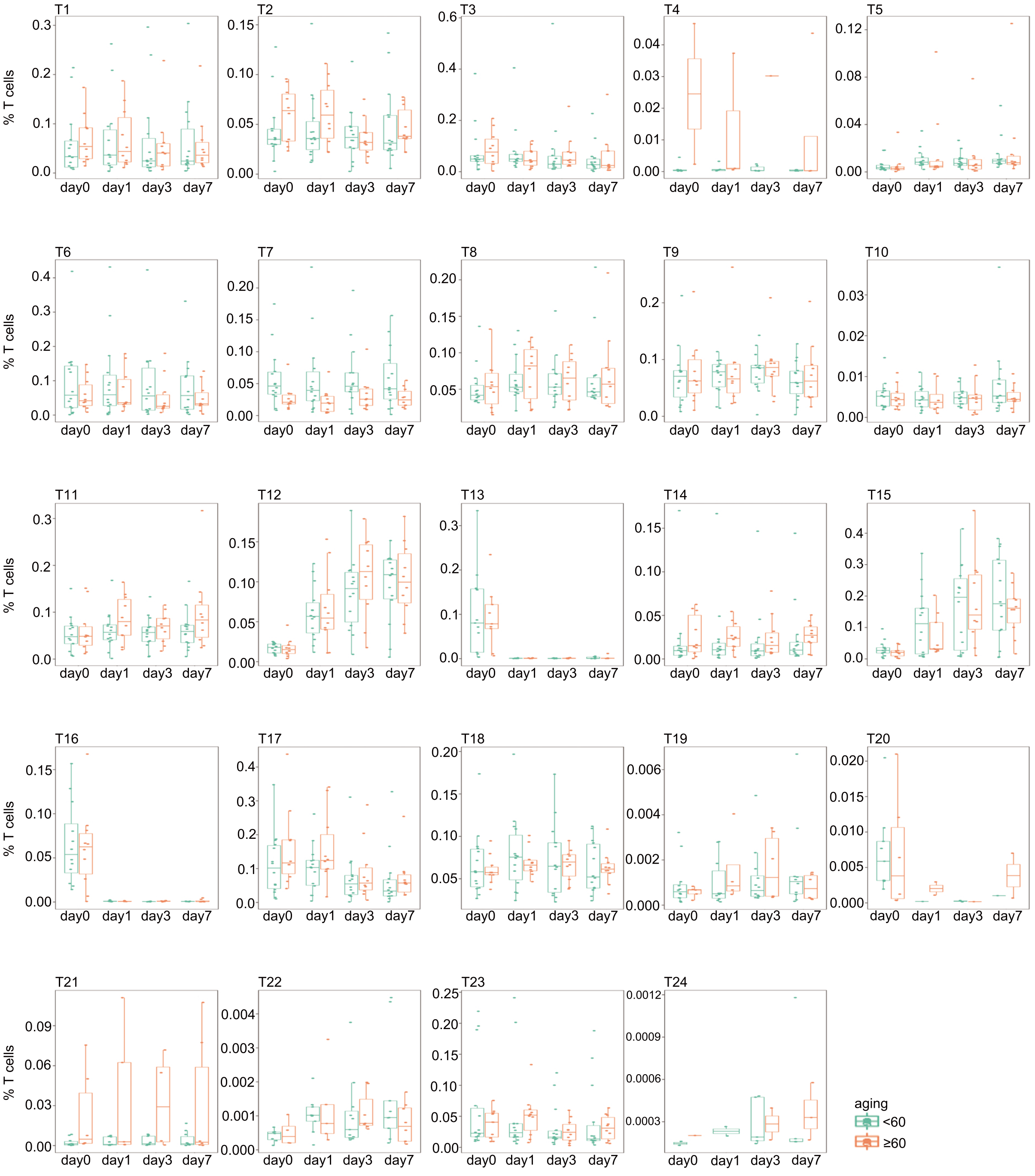

Supplementary Figure 15

A

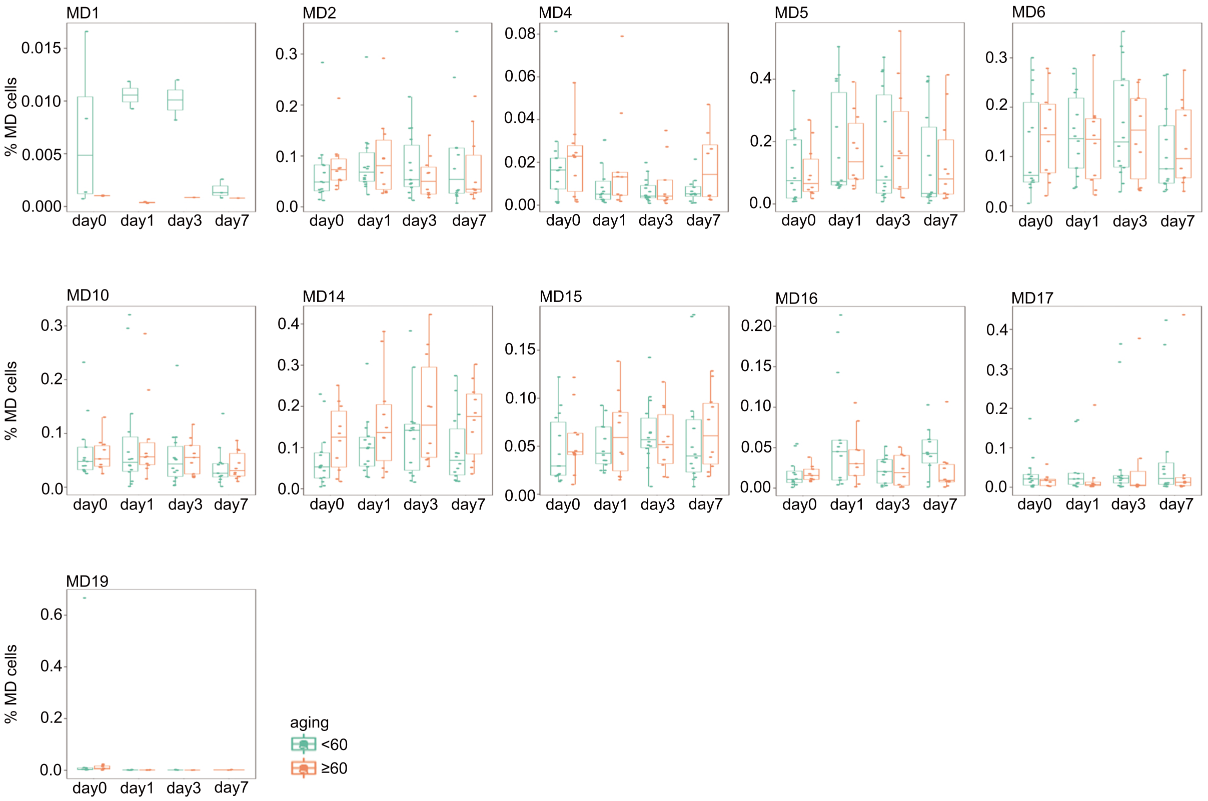

B

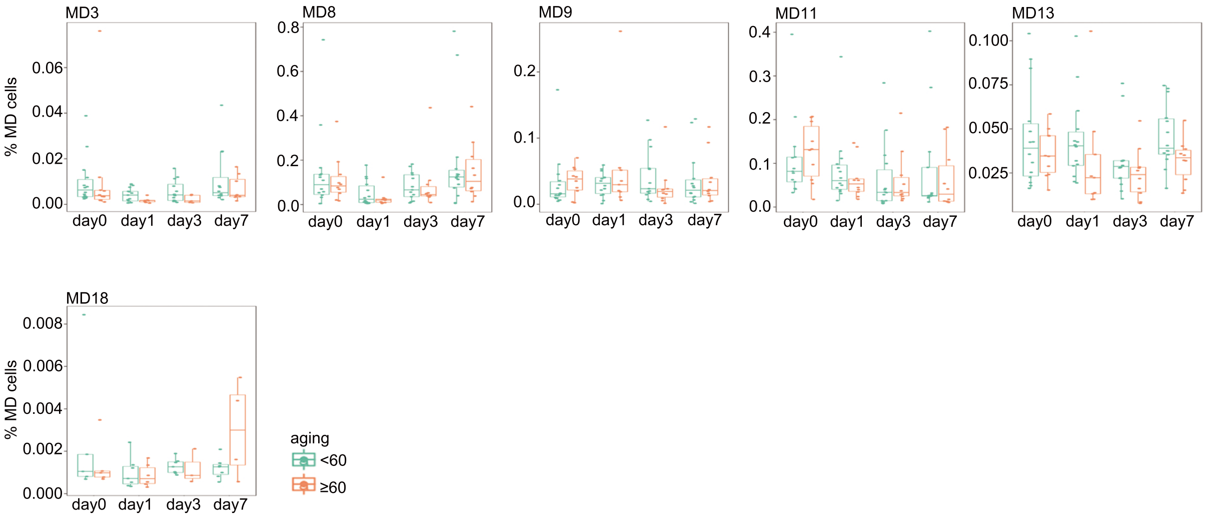

Supplementary Figure 16

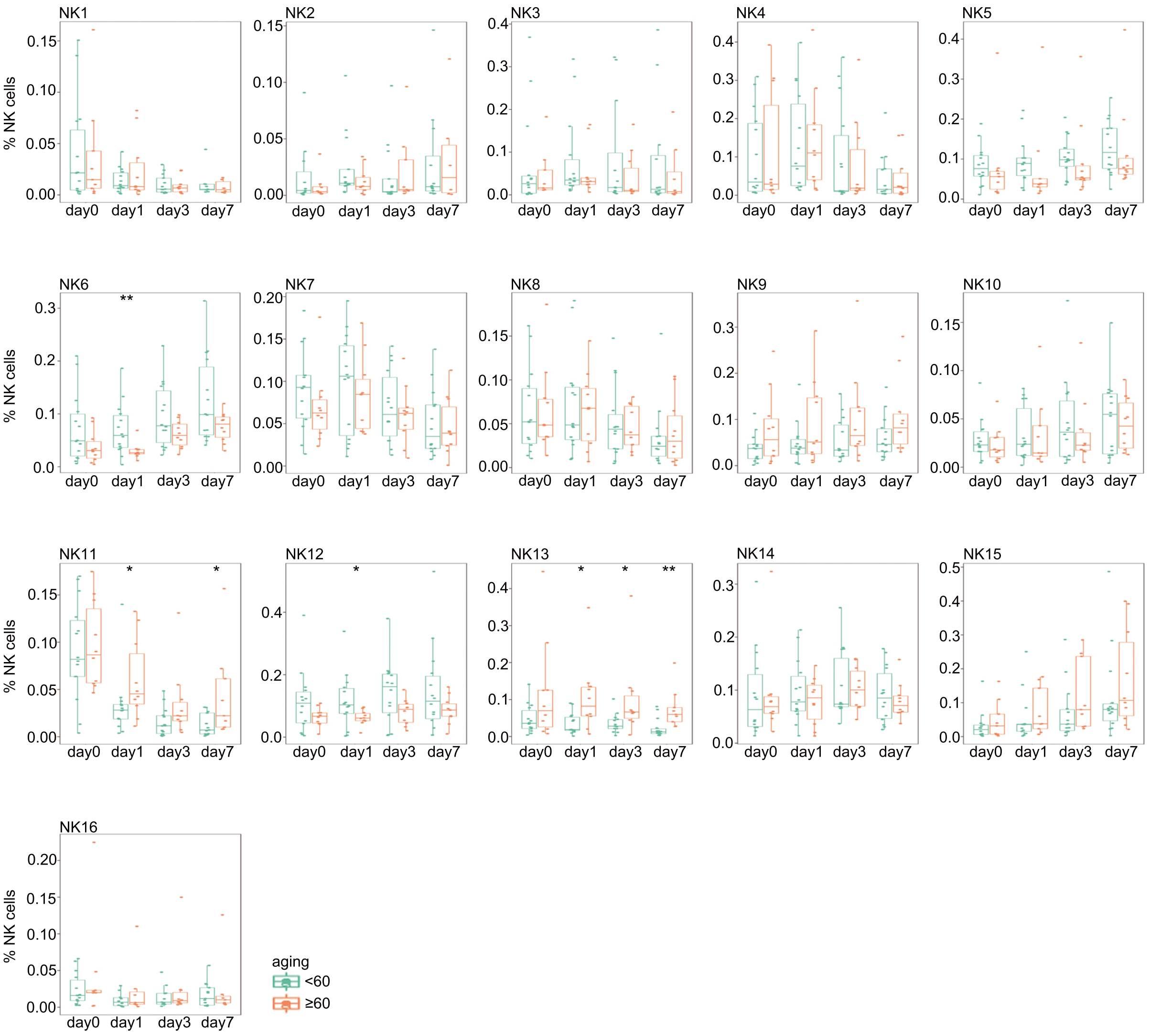

Supplementary Figure 17

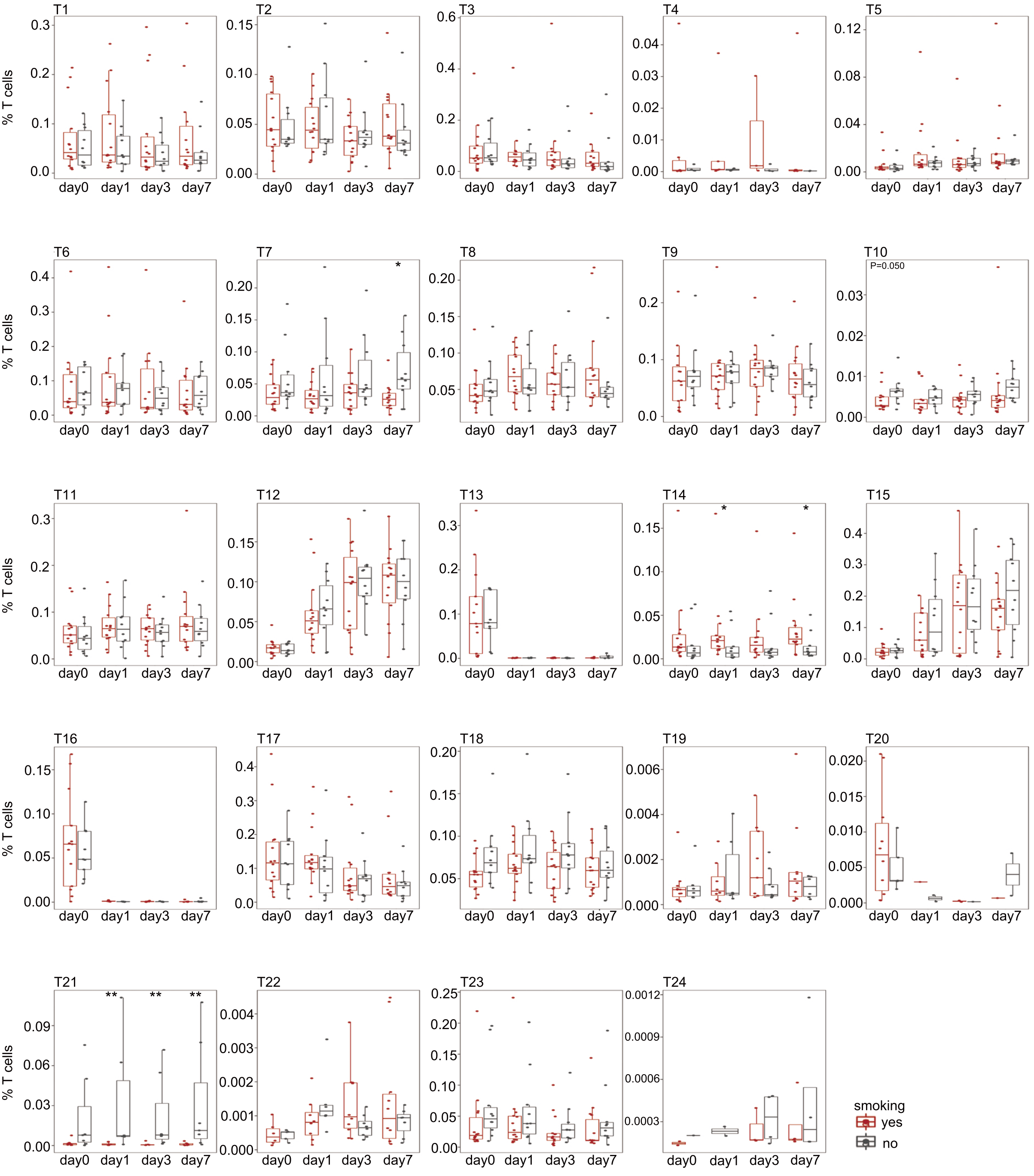

Supplementary Figure 18

A

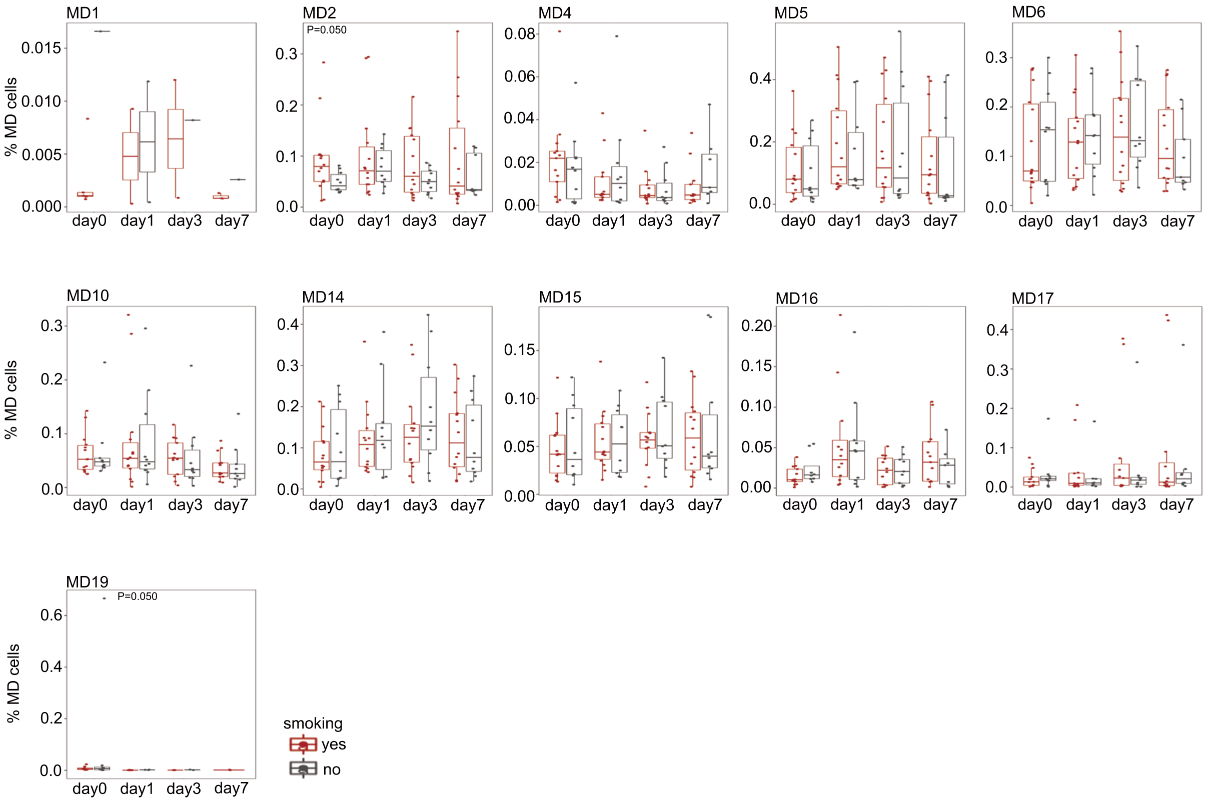

B

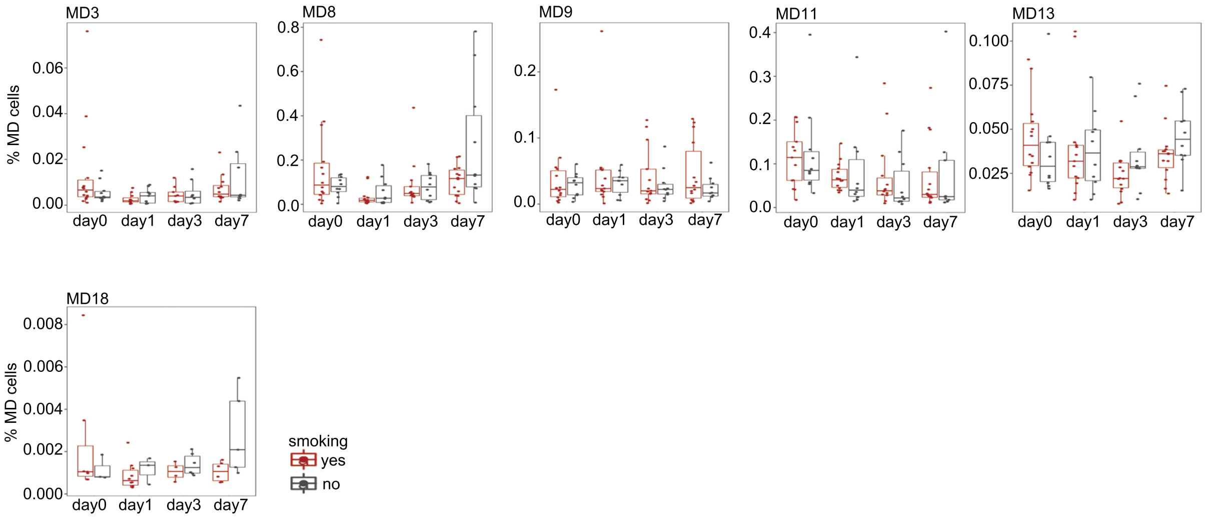

Supplementary Figure 19

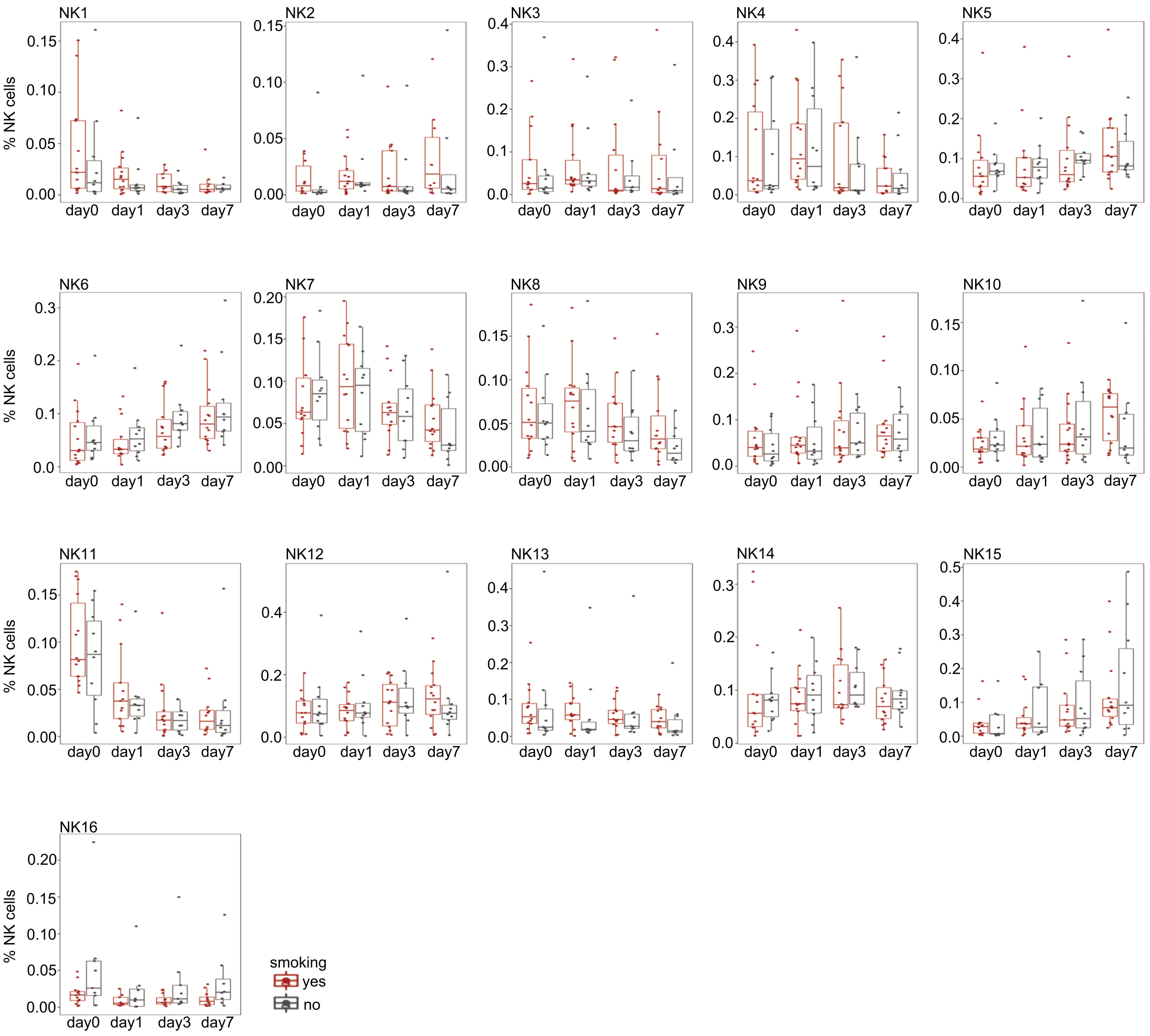

Supplement: Supplementary file 1 [file DataSheet_1.zip › Data_Sheet_1.pdf]
